# The role of genomic location and flanking 3′UTR in the generation of functional levels of variant surface glycoprotein in *Trypanosoma brucei*

**DOI:** 10.1111/mmi.13838

**Published:** 2017-10-11

**Authors:** Sophie Ridewood, Cher‐Pheng Ooi, Belinda Hall, Anna Trenaman, Nadina Vasileva Wand, Georgios Sioutas, Iris Scherwitzl, Gloria Rudenko

**Affiliations:** ^1^ Department of Life Sciences, Sir Alexander Fleming Building Imperial College London South Kensington London SW7 2AZ UK

## Abstract

*Trypanosoma brucei* faces relentless immune attack in the mammalian bloodstream, where it is protected by an essential coat of Variant Surface Glycoprotein (VSG) comprising ∼10% total protein. The active *VSG* gene is in a Pol I‐transcribed telomeric expression site (ES). We investigated factors mediating these extremely high levels of VSG expression by inserting ectopic *VSG117* into VSG221 expressing *T. brucei*. Mutational analysis of the ectopic *VSG* 3′UTR demonstrated the essentiality of a conserved 16‐mer for mRNA stability. Expressing ectopic *VSG117* from different genomic locations showed that functional VSG levels could be produced from a gene 60 kb upstream of its normal telomeric location. High, but very heterogeneous levels of VSG117 were obtained from the Pol I‐transcribed rDNA. Blocking VSG synthesis normally triggers a precise precytokinesis cell‐cycle checkpoint. VSG117 expression from the rDNA was not adequate for functional complementation, and the stalled cells arrested prior to cytokinesis. However, VSG levels were not consistently low enough to trigger a characteristic ‘VSG synthesis block’ cell‐cycle checkpoint, as some cells reinitiated S phase. This demonstrates the essentiality of a Pol I‐transcribed ES, as well as conserved *VSG* 3′UTR 16‐mer sequences for the generation of functional levels of VSG expression in bloodstream form *T. brucei*.

## Introduction

The African trypanosome *Trypanosoma brucei* is a paradigm for monoallelic control and antigenic variation. *T. brucei* is the causative agent of Human African Trypanosomiasis and ‘nagana’ in livestock, which are transmitted by tsetse flies. Although case numbers for trypanosomiasis have been falling, 70 million people are still estimated to be at potential risk of infection (Franco *et al*., 2014). In addition to the human mortality, livestock diseases caused by *T. brucei* and related trypanosomatids cause enormous economic losses. It has been estimated that eliminating these would result in a net benefit to African countries of nearly $2.5 billion over a 20 year period (Shaw *et al*., 2014). *T. brucei* thrives in the bloodstream of the mammalian host, despite being exposed to continuous attack by components of the immune system including complement and antibodies (Rudenko, 2011; Mugnier *et al*., 2016). A chronic infection is maintained through a highly sophisticated strategy of antigenic variation, based on the monoallelic expression of Variant Surface Glycoprotein (VSG) (Glover *et al*., 2013b; Gunzl *et al*., 2015; Duraisingh and Horn, 2016).

Individual parasites are coated with a VSG coat composed of a dense layer of 10 million rod‐like VSG molecules attached to the *T. brucei* cell surface via a glycosylphosphatidylinositol (GPI) anchor (Cross, 1975; Schwede and Carrington, 2010; Schwede *et al*., 2015). VSG is the most abundant protein in bloodstream form *T. brucei* making up about 10% of the total protein (Wang *et al*., 2003), and is essential even *in vitro*. Blocking VSG synthesis triggers a cell‐cycle checkpoint resulting in cells stalled pre‐cytokinesis which do not undergo re‐initiation of S‐phase (Sheader *et al*., 2005; Smith *et al*., 2009).

The gene for the active VSG is located in one of about fifteen extensive (40–60 kb) telomeric *VSG* expression site (ES) transcription units containing large families of polymorphic expression site associated genes as well as the telomeric *VSG* (Becker *et al*., 2004; Hertz‐Fowler *et al*., 2008). ESs are controlled in a strictly mono‐allelic fashion (Chaves *et al*., 1999; Glover *et al*., 2016). Although the GPI‐anchored VSG protein is highly immunogenic, stochastic VSG switch events occur in the population, leading to the expression of new and immunologically distinct variants. VSG switching can involve a transcriptional switch to another ES (Alsford *et al*., 2012). Alternatively, movement of silent *VSG*s (or segments of *VSGs*) into the active ES through DNA rearrangements allows the trypanosome to switch between different VSGs or create new ‘mosaic’ variants (Hall *et al*., 2013; McCulloch *et al*., 2015). As *T. brucei* has an extensive wardrobe of thousands of *VSG* genes and pseudogenes, a chronic infection can be mounted which can last for years (Marcello and Barry, 2007; Cross *et al*., 2014; Mugnier *et al*., 2015).

Unusually, *VSG* ESs are transcribed by RNA polymerase I (Pol I), which exclusively transcribes ribosomal DNA (rDNA) in other eukaryotes (Gunzl *et al*., 2003). The active ES is located in a non‐nucleolar location known as the Expression Site Body (ESB) (Navarro and Gull, 2001). The ESB is formed around a transcriptionally active ES (Kerry *et al*., 2017), and presumably contains the transcription and RNA processing machinery necessary for the production of very high levels of *VSG* transcript. *T. brucei* is the only eukaryote known to be capable of utilising Pol I to transcribe protein coding genes, including those encoded in ESs as well as procyclin (major surface protein of procyclic *T. brucei*) (Roditi *et al*., 1998; Gunzl *et al*., 2003). This unusual ability is presumably possible because trans‐splicing adds a capped Pol II‐derived spliced leader RNA to the Pol I‐derived transcript which would otherwise be uncapped, and, therefore, untranslatable (Bruderer *et al*., 2003).

All other protein coding genes in *T. brucei* are present in extensive polycistronic arrays which are constitutively transcribed by Pol II (Kolev *et al*., 2010). There is no evidence for regulated Pol II transcription, and Pol II promoter elements appear to be simple G‐stretches, which are functionally defined predominantly at the epigenetic level (Siegel *et al*., 2009; Wright *et al*., 2010). High levels of gene expression in *T. brucei* can be a consequence of gene amplification, with some particularly abundant proteins encoded by large gene families (Berriman *et al*., 2005). However, RNA levels are predominantly modulated post‐transcriptionally through RNA stability elements, allowing life‐cycle specific expression of constitutively transcribed genes (Kramer, 2012; Clayton, 2014).

For antigenic variation to work effectively, there needs to be mono‐allelic expression of a single surface antigen type, and the major variant needs to be continuously switched during a chronic infection. The trypanosome is, therefore, restricted to express the vast amount of VSG it requires from a single copy gene. We asked which features allow the trypanosome to generate such high levels of VSG expression from a single gene, and tested the functionality of ectopic *VSG* located in different genomic locations, and flanked downstream by different 3′ untranslated regions (UTRs). We demonstrate the essentiality of a conserved 16‐mer sequence within the *VSG* 3′UTR for conferring functional levels of *VSG* mRNA stability (Berberof *et al*., 1995). In addition, we show that high levels of VSG are only expressed from Pol I‐ transcribed loci, although functional levels of VSG expression were only obtained from the Pol I‐transcribed ES. The exact location of the *VSG* gene within the ES transcription unit was not critical. In contrast, the level of VSG expression from the Pol I‐transcribed rDNA loci was heterogeneous, and did not adequately complement the cell when endogenous *VSG* transcript was knocked‐down using RNAi. These results highlight key features essential for generating functional levels of VSG expression in bloodstream form *T. brucei*, enabling it to be such an effective pathogen.

## Results

### Functional levels of VSG can be expressed 60 kb upstream of the ES telomere

VSG is the most abundant protein in bloodstream form *T. brucei*, and a relatively minor reduction in its expression level is detrimental even *in vitro* in the absence of an immune system (Sheader *et al*., 2005). The active *VSG* is expressed from an extensive (40–60 kb) telomeric bloodstream form ES, where it is invariably located adjacent to the telomere repeats (Berriman *et al*., 2002; Becker *et al*., 2004; Hertz‐Fowler *et al*., 2008). We first investigated the role of genomic location in facilitating these very high levels of VSG expression, and determined if functional levels could be expressed from a *VSG* located immediately downstream of the active ES promoter, rather than at its normal telomeric location 60 kb downstream.

**Figure 1:**
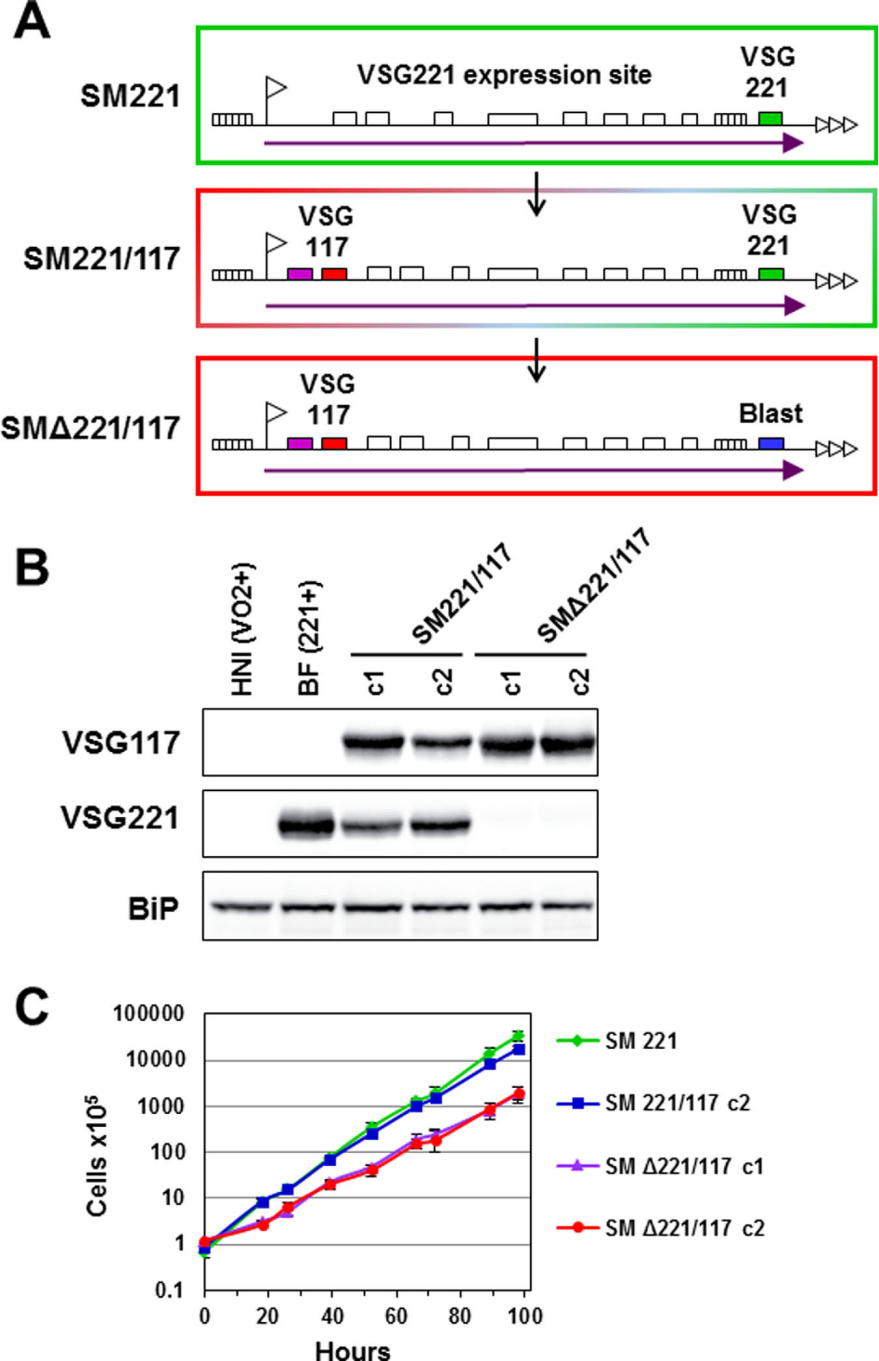
Functional levels of VSG expression from a gene inserted 60 kb upstream of the ES telomere. A. Schematic showing the generation of the *T. brucei* SM221/117 ‘double‐expresser’ cell line and the subsequent deletion of telomeric *VSG221* to generate SMΔ221/117. *T. brucei* SM221 expresses VSG221 from the active *VSG221* ES, where the ES promoter is indicated with a flag, relevant ES associated genes with open boxes, simple sequence repeats with hatched boxes, telomere repeats with horizontal arrows, and ES transcription with an arrow. A construct containing *VSG117* (red box) and a puromycin resistance gene (purple box) was integrated immediately downstream of the *VSG221* ES promoter, generating SM221/117. The *VSG221* gene (green box) was subsequently deleted and replaced with a blasticidin resistance gene (blue box), generating SMΔ221/117. B. Western blot analysis of VSG221 and VSG117 expression in the *T. brucei* SM221/117 or SMΔ221/117 cell lines, where two clones (c1 and c2) were analysed for each. The VSGVO2 expressing cell line *T. brucei* HNI(VO2+) and the VSG221 expressing cell line *T. brucei* BF(221+) are included as controls, and BiP protein served as a loading control. C. Cumulative growth curve of the parental *T. brucei* SM221 cell line, one clone of *T. brucei* SM221/117 (c2), and two clones of *T. brucei* SMΔ221/117 (c1 and c2). The mean of three replicates is shown with standard deviation indicated with error bars.

We inserted a construct containing ectopic *VSG117* immediately downstream of the active *VSG221* ES promoter in the *T. brucei* 427 ‘single‐marker’ cell line (SM221) (Fig. 1A) (Wirtz *et al*., 1999; Smith *et al*., 2009). The resulting *T. brucei* SM221/117 cell line expressed high levels of ectopic VSG117 in a background of endogenous VSG221. This resulted in a reduction in VSG221 to approximately 50% wild type levels. Although the relative ratios of expression of VSG117 and VSG221 were variable, the levels appeared inversely correlated with each other (Fig. 1B). This pattern of an approximately inverse correlation between the amount of ectopic *VSG117* and endogenous *VSG221* mRNA within the cell was repeatedly observed throughout this study using a broad range of different cell lines (Supporting Information Fig. S1).

The growth rate of *T. brucei* ‘double‐expressers’ expressing two *VSG*s from the same ES was the same as that of *T. brucei* expressing only *VSG221* (Fig. 1C) (Munoz‐Jordan *et al*., 1996; Smith *et al*., 2009). Blocking VSG synthesis with RNAi leads to an abrupt cell‐cycle arrest, which can be rescued by expression of a second ectopic *VSG* from within the active ES (Smith *et al*., 2009). However, as RNAi does not result in complete removal of all targeted mRNA, we asked in a more rigorous fashion if bloodstream form *T. brucei* could be made fully reliant on a *VSG* expressed from immediately behind the ES promoter more than 60 kb upstream of its normal location.

We, therefore, deleted the telomeric *VSG221* in these ‘double‐expresser’ *T. brucei* and replaced it with a blasticidin resistance gene (Fig. 1A). The resulting ‘single‐expresser’ *T. brucei* SMΔ221/117 cell line only expressed VSG117 (Fig. 1B), at levels which were increased compared with ‘double‐expresser’ cells. This indicates that a fixed maximal amount of VSG can be stably expressed, which is also essential for trypanosome survival. The VSG117 ‘single‐expresser’ cell lines grew slightly slower than either the single or double‐expresser parental lines (Fig. 1C). However, this could be a consequence of slightly less optimal levels of VSG117 expression, possibly due to suboptimal RNA processing signals around the ectopic *VSG117*. As ectopic expression of *VSG117* could rescue the cell from a precytokinesis arrest, this demonstrates that the invariably telomeric location of *VSG* within the bloodstream form ES is not essential for adequate levels of expression.

### High levels of expression of ectopic VSG117 from Pol l transcribed loci

**Figure 2:**
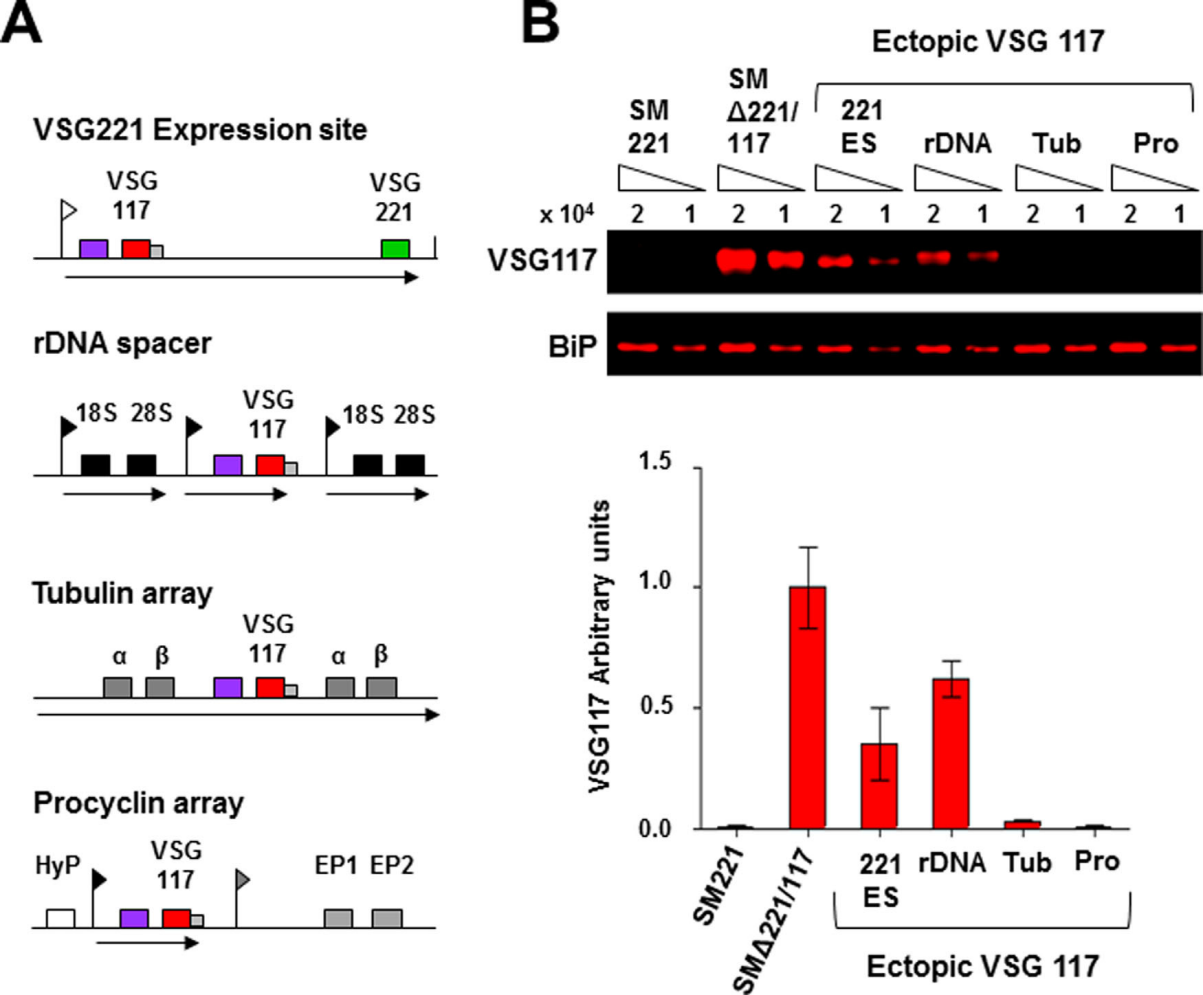
High levels of VSG expression can only be obtained from Pol I transcription units. A. Schematic showing the integration of constructs containing *VSG117* (red box) and a hygromycin resistance gene (violet box) into different genomic loci in *T. brucei* expressing VSG221. These include immediately downstream of the *VSG221* ES promoter (white flag), within a tagged rDNA spacer (18S and 28S rRNA genes indicated with black boxes), within the Pol II‐transcribed αβ tubulin array (genes indicated with grey boxes) or upstream of the silent procyclin transcription units (hypothetical protein indicated with HyP, procyclin promoter with a grey flag, and EP1 and EP2 procyclin genes with grey boxes). Constructs integrating into the rDNA spacer or upstream of the procyclin genes contain an ectopic rDNA promoter (black flag) directing transcription. Transcription is indicated with arrows. B. Levels of VSG117 expressed from ectopic loci assayed using LiCor analysis. VSG221 is expressed from the active *VSG221* ES in all cell lines except for SMΔ221/117. Quantification is shown below, with data presented as arbitrary units normalised to BiP. Results are from three biological replicates, with standard deviation indicated with error bars

We next expressed ectopic *VSG117* from other genomic locations, including a tagged Pol I rDNA spacer, a Pol II‐transcribed α‐β tubulin locus, or upstream of a silent Pol I procyclin transcription unit (Fig. 2A), where constructs integrating in the rDNA spacer or upstream of the procyclin genes were transcribed by an ectopic rDNA promoter. Levels of ectopically expressed VSG117 were quantified using LiCor analysis (Fig. 2B). As seen previously, expression of ectopic *VSG117* in a background of endogenous *VSG221* appeared to result in a reduction in VSG117 levels compared with VSG117 ‘single‐expresser’ *T. brucei*. High levels of VSG117 were expressed from either the Pol I‐transcribed ES or rDNA (at respectively 34.3 ± 17.9% or 62.4 ± 8.4% levels of the VSG117 ‘single‐expresser’ *T. brucei* SMΔ221/117). In contrast, very low levels of VSG117 expression were obtained from the Pol II α‐β tubulin transcription unit (2.9 ± 0.7%), and negligible amounts (< 1%) were obtained upstream of the inactive procyclin loci.

**Figure 3:**
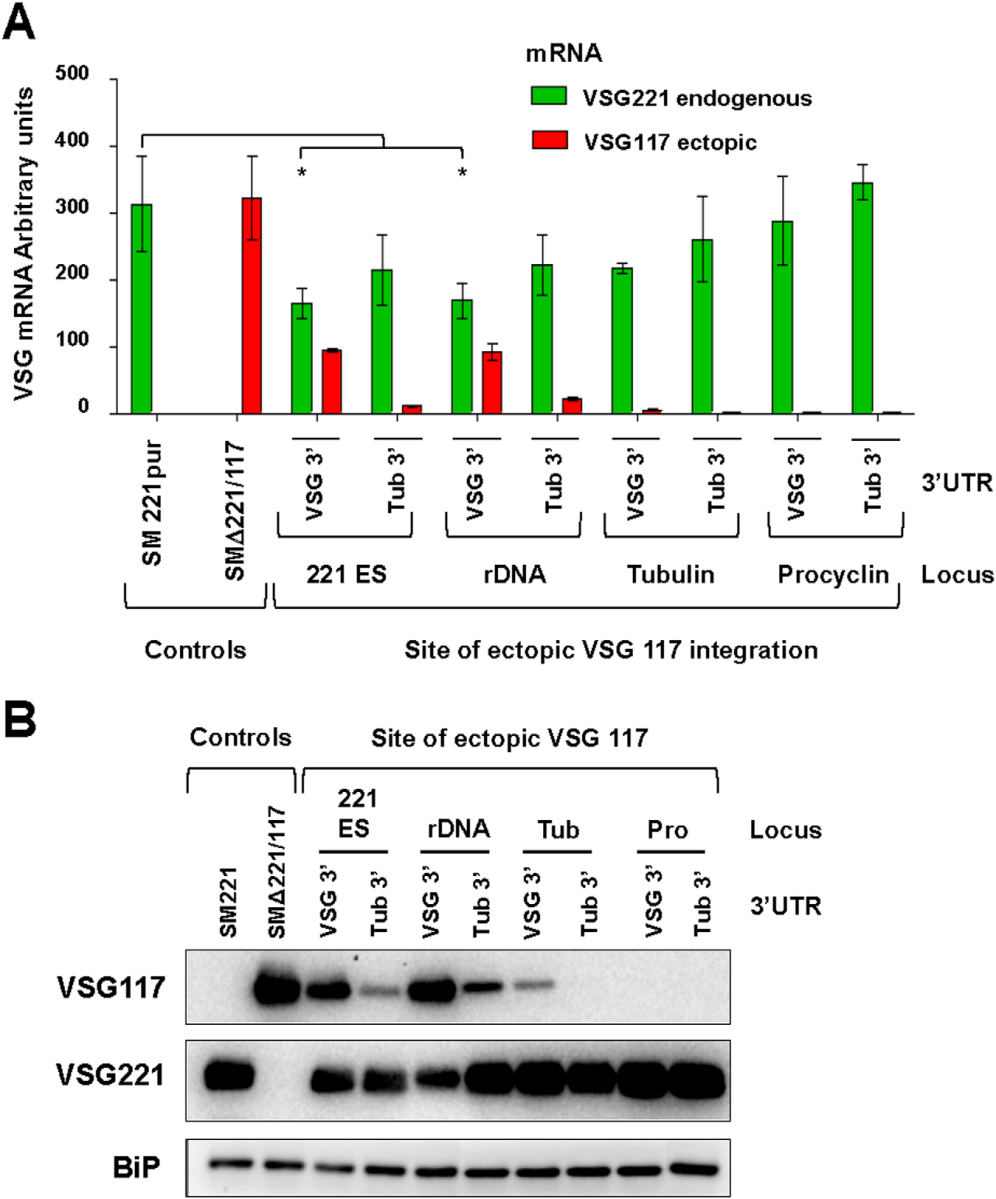
The *VSG* 3′UTR is essential for high levels of expression of *VSG* mRNA. A. Quantification of mRNA levels expressed from ectopic *VSG117* or endogenous *VSG221* using qPCR in the cells lines shown in Fig. 2A. Ectopic *VSG117* was expressed from immediately behind the *VSG221* ES promoter, in the rDNA spacer, the tubulin array or upstream of the procyclin loci. *VSG117* was flanked downstream by either a *VSG221* (VSG 3′) or tubulin (Tub 3′) UTR. The VSG221 or VSG117 single expressers (SM221pur or SMΔ221/117 respectively) are shown as controls. *VSG* transcript levels were normalised to actin. There was significant downregulation of endogenous *VSG221* transcript in cells expressing high levels of *VSG117* from either the *VSG221* ES or the rDNA spacer (* *P* < 0.05). The results are from three biological replicates with the standard deviation indicated with error bars. B. Western blot analysis of VSG117 expressed ectopically from the *VSG221* ES, the rDNA locus, tubulin locus (tub) or upstream of the procyclin loci (Pro). *VSG117* was flanked downstream with a *VSG221* or a tubulin 3′UTR. The VSG221 or VSG117 single expressers (SM221 or SMΔ221/117 respectively) are included for comparison. The blot was probed for VSG117 or VSG221 with BiP used as a loading control.

We next determined the role of the *VSG* 3′ flanking regions for VSG expression, and compared levels of expression of ectopic *VSG117* flanked downstream by 3′ sequences from either *VSG221* or α‐tubulin (Fig. 3). High levels of VSG117 expression could only be obtained if the *VSG* gene was flanked downstream by a *VSG* 3′ sequence. Replacing these 3′ flanking sequences with those from tubulin resulted in a 4–12‐fold reduction in *VSG117* transcript levels (Fig. 3A). Negligible amounts of ectopic *VSG117* were expressed from either the αβ‐tubulin array or upstream of the procyclin transcription unit irrespective of the 3′ flanking region. These results were also reflected at the protein level (Fig. 3B). The higher levels of VSG117 expression obtained from a *VSG117* gene flanked with *VSG* 3′ sequences was a consequence of a stabilising effect of these sequences on the *VSG* transcript. *VSG* mRNA undergoes biphasic RNA decay, characterised by initial slow decay followed by a faster reduction in transcript levels (Hoek *et al*., 2002; Clayton, 2014). This appears to be a general feature for *T. brucei* mRNAs that have long half‐lives (Fadda *et al*., 2014). *VSG117* transcript with *VSG* 3′ sequences had a mRNA half‐life of 88.7 ± 22.4 min, compared with 29.7 ± 11.4 min if these 3′ sequences were replaced with those from tubulin (Supporting Information Fig. S2). Our estimate of a *VSG* mRNA half‐life of between 85 and 99 min is comparable with the value of 84–90 min previously determined using Northern blotting (Hoek *et al*., 2002).

We next sought to determine if the adjacent regions downstream of the *VSG* had an effect on the increased RNA stability, and found that the observed stabilising effect on the *VSG* mRNA transcript was due to the *VSG* 3′UTR rather than the adjacent 3′ flanking regions. We analysed trypanosomes expressing ectopic *VSG117* flanked with chimeric *VSG* and α‐tubulin 3′ downstream sequences (Supporting Information Figs S3 and S4). High levels of expression of ectopic *VSG117* were only obtained if *VSG117* was flanked with a *VSG* 3′UTR, even if the regions downstream of the polyadenylation site were switched for those downstream of an α‐tubulin polyadenylation site (Supporting Information Figs S3 and S4). *T. brucei* genes tend to show variability in the polyadenylation sites used (Siegel *et al*., 2010). Using Rapid Amplification of 3′ Ends (3′RACE) (Scotto‐Lavino *et al*., 2006) we found that the preferred *VSG* polyadenylation site did not change in these chimeric 3′ sequences, although there was an increase in use of alternative *VSG* polyadenylation sites (Supporting Information Fig. S4 and Table S1). This demonstrates that the *VSG* 3′UTR was the main factor in facilitating high levels of expression of ectopic *VSG117*, and that this was a consequence of a transcript stabilising effect of the *VSG* 3′UTR (Berberof *et al*., 1995).

As shown earlier, expression of a higher level of an ectopic *VSG117* resulted in a compensatory decrease in levels of expression of the endogenous *VSG221*, which was seen at both the RNA and protein level (Fig. 3) (Supporting Information Fig. S1). Decrease in endogenous *VSG221* transcript was significant and highly repeatable (* *P* < 0.05). It has been previously documented that expression of an ectopic *VSG* from a tetracycline inducible T7 promoter leads to transcriptional attenuation of the active ES telomere (Batram *et al*., 2014). However, this attenuation is unstable, and disappears within days. We did not find evidence for significant transcriptional attenuation of the *VSG221* ES telomere when an ectopic *VSG117* was expressed from a location either immediately proximal to the active *VSG221* ES promoter, or from the rDNA spacer.

**Figure 4:**
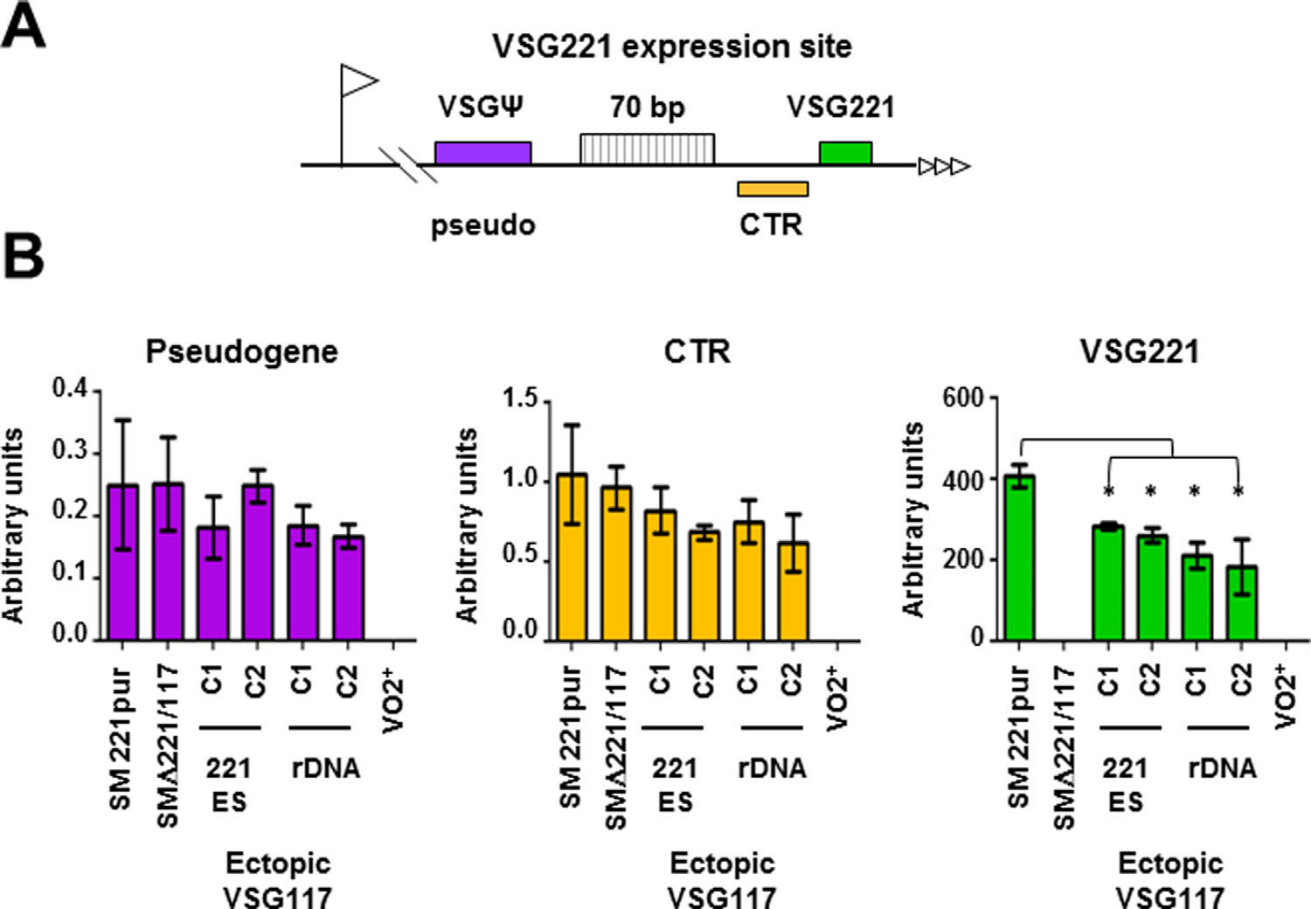
Expression of ectopic *VSG117* leads to significant reduction in levels of *VSG221* mRNA but no significant attenuation of transcription at the active *VSG221* telomere. A. Schematic of the *VSG221* ES telomere indicating the relative positions of a single copy *VSG* pseudogene (ψ), 70 bp repeats, the *VSG221* co‐transposed region (CTR) and *VSG221*. The ES promoter is indicated with a flag and telomere repeats with horizontal arrows. The schematic is not to scale. B. Quantification of RNA transcript levels corresponding to the *VSG* pseudogene, CTR or *VSG221* in cells where ectopic *VSG117* with a *VSG221* 3′UTR was inserted into the active *VSG221* ES or an rDNA spacer using qPCR. Transcript levels were normalised against actin, and data are presented as arbitrary units (2^–ΔCt^). The ‘single‐expresser’ SM221pur (221+) and SMΔ221/117 (117+) cell lines, as well as the HNI(VO2) cell line (V02+) with an active *VSGV02* ES are included as controls. As expected, there was significant reduction in levels of *VSG221* transcript on expression of ectopic *VSG117* (* *P* < 0.05, one way ANOVA and Tukey *post hoc*). However, there was no significant reduction in the level of other precursor RNAs derived from the *VSG221* ES telomere. Results were derived from three biological replicate experiments with standard deviation indicated with error bars.

The *VSG221* ES telomere contains unique sequences including a single copy *VSG* pseudogene (pseudo 1.10100, and the *VSG221* co‐transposed region (CTR) (Fig. 4A) (Davies *et al*., 1997). Unstable transcripts are generated from these sequences. We saw a significant reduction in *VSG221* transcript when ectopic *VSG117* was expressed from either the ES or the rDNA spacer (Fig. 4B) (*P* < 0.05). Quantification of the low abundance transcripts from the *VSG* pseudogene or the CTR gave a higher degree of variability than quantification of *VSG221* mRNA. However, we did not find evidence for a statistically significant reduction in their abundance in the presence of expression of ectopic *VSG117* (Fig. 4B). This absence of significant transcriptional attenuation argues that the inverse correlation in the amounts of VSG117 and VSG221 that we observe (Supporting Information Fig. S1) is operating at the level of mRNA stability, and could be the consequence of a limiting, and transcript stabilising *VSG* 3′UTR binding protein.

### A highly conserved 16‐mer in the VSG 3′ UTR is essential for mRNA stability

**Figure 5:**
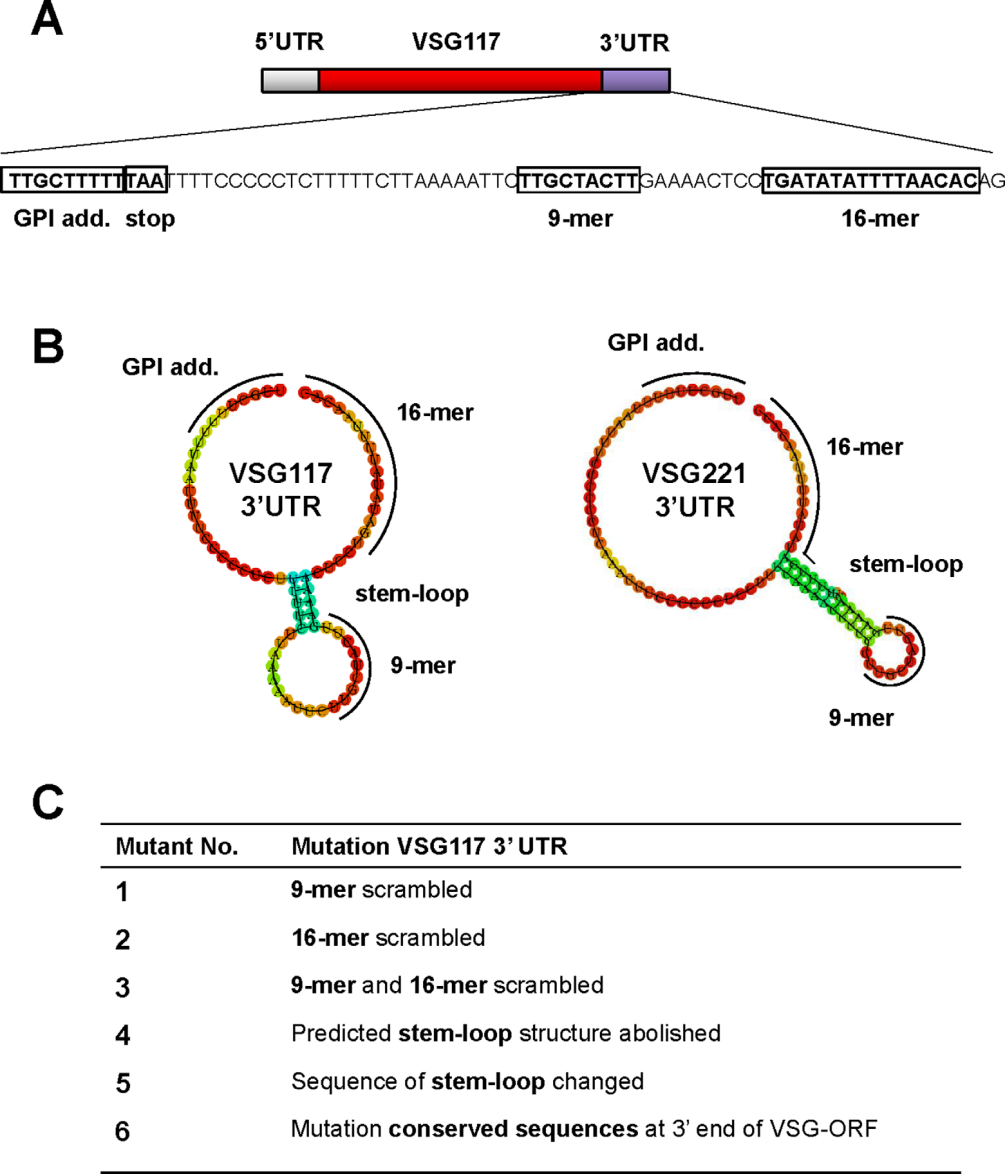
Mutational analysis of highly conserved features of the *VSG* 3′ UTR. A. Schematic of the *VSG117* gene with untranslated regions (UTR) and open reading frame (ORF) indicated. Sequence of the *VSG117* 3′ UTR is shown with relevant sequences indicated with boxes including the GPI anchor addition sequence (GPI add.), the stop codon, and sequences that are highly conserved in functional *VSG* transcripts: the 9‐mer and the 16‐mer. In addition, a highly conserved sequence at the 3′ end of the *VSG* ORF is shown which encodes a Leu‐Leu dipeptide which is part of the GPI anchor addition signal. B. Predicted RNA secondary structure of the *VSG117* or *VSG221* 3′ UTR as determined using RNAfold (ViennaRNA Package 2.0). The positions of relevant features in the predicted RNA structures are highlighted with black lines. The colours of the dots indicate the probability of RNA nucleotides being unpaired from unlikely (blue = 0) to highly likely (red = 1). C. Overview of six *VSG117* 3′ UTR mutations generated to probe the essentiality of specific highly conserved sequences, as well as predicted *VSG* UTR secondary structure. Mutations 1–3 scrambled the sequences of the conserved 9‐mer and/or 16‐mer. Mutations 4 and 5 either abolished the stem‐loop structure, or maintained its structure but changed its sequence. Mutation 6 mutated a conserved sequence within the *VSG117* ORF without disrupting the GPI anchor addition sequence. The sequences of the *VSG* 3′ UTR mutations analysed are in the Supporting Information.

Alignment of the *VSG* 3′ UTR in 31 *VSG* cDNA sequences identified highly conserved 9‐mer and 16‐mer sequences (Fig. 5A, Supporting Information Fig. S5) (Borst and Cross, 1982; Berberof *et al*., 1995; Hutchinson *et al*., 2007). We determined the predicted secondary structure of these *VSG* 3′UTRs using RNAfold (ViennaRNA Package 2.0) (Gruber *et al*., 2008), and found that most *VSG* 3′ UTR sequences form a hairpin with the 9‐mer located in the loop (Fig. 5B). We, therefore, used mutational analysis to test the functional role of these different *VSG* 3′UTR sequences and hypothetical RNA folding structures. We expressed ectopic *VSG117* flanked downstream with a *VSG117* 3′UTR with scrambled 9‐mer and/or 16‐mer sequences (Fig. 5C). In addition, we created mutations where we abolished the predicted stem‐loop structure or recreated it using different sequences. Last, we mutated conserved sequences at the 3′ end of the *VSG* open reading frame (Fig. 5C) (Supporting Information Table S2).

**Figure 6:**
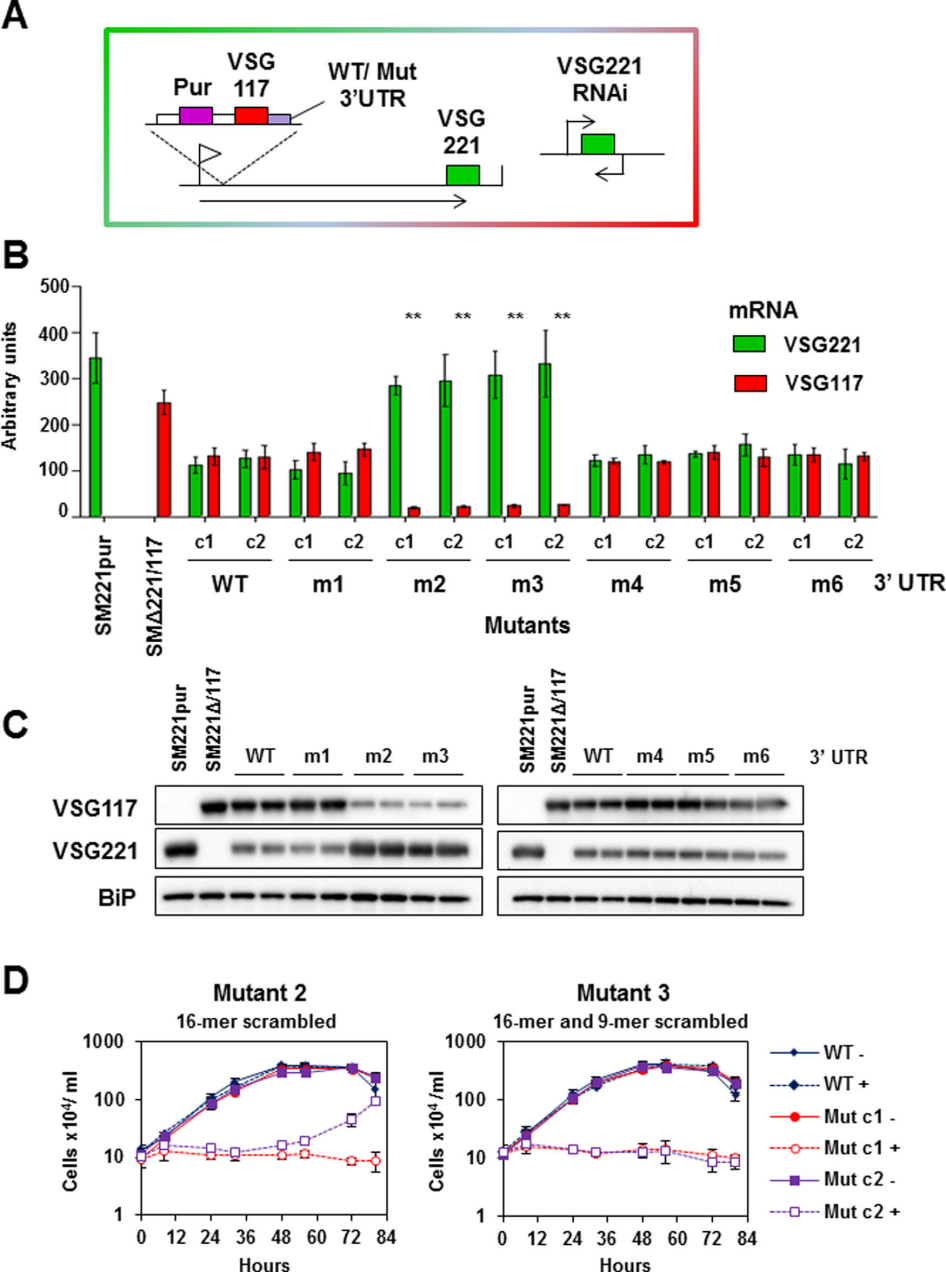
The conserved 16‐mer sequence within the *VSG* 3′UTR is essential for high levels of VSG expression. A. Schematic of the cell line used for the *VSG* 3′UTR studies. Constructs containing *VSG117* flanked downstream by either wild type (WT) or mutant *VSG117* 3′ UTRs were inserted into the active *VSG221* expression site (ES) of the bloodstream form *T. brucei* 221VB1.1 cell line. The selectable marker was a puromycin (Pur) resistance gene. *VSG221* RNAi can be induced in this cell line, which allows one to establish if ectopic *VSG117* can compensate for lack of *VSG221* transcript. Promoters are indicated with flags and transcription with arrows. B. Levels of *VSG117* and *VSG221* transcript as determined using qPCR. RNA was isolated from cell lines expressing ectopic *VSG117* flanked downstream by either a wild type *VSG117* UTR, or a *VSG117* UTR with mutations in conserved features. The *VSG* 3′UTR mutant numbers (1–6) are described in Fig. 5C. RNA from the SM221pur (221+) or SMΔ221/117 (117+) ‘single‐expresser’ strains is analysed as a control. Values normalised against actin are shown in arbitrary units (2^‐ΔCt^), with the amount of *VSG221* (red bars) or *VSG117* (green bars) transcript shown as the mean of three independent experiments with the standard deviation indicated with error bars. The only significant reduction in *VSG117* transcript compared with wild type (WT) was in mutants m2 and m3 containing scrambled 16‐mer sequences (** *P* < 0.01). C. Western blot analysis of VSG levels in *T. brucei* expressing ectopic *VSG117* with either a wild type (WT) or mutant *VSG117* 3′ UTRs (m1–m6). Protein lysates were isolated from the cell lines analysed in panel B. Protein from two clones of each mutant are shown in comparison with ‘single‐expresser’ cell lines SM221pur (VSG221+) or SMΔ221/117 (VSG117+). VSG117 or VSG221 were probed for, with BiP serving as a loading control. D. The conserved 16‐mer present in the *VSG* 3′UTR is required for functional levels of VSG expression. Growth curves were performed in VSG221 expressing *T. brucei* cell lines expressing ectopic *VSG117* with either a wild type (WT) or a mutated *VSG* 3′UTR (Mutant 2 or Mutant 3). *VSG221* RNAi was induced in the presence (+) or absence (‐) of tetracycline. Two clones are shown for each mutant (c1 and c2). Results are the mean of three independent experiments with standard deviation indicated with error bars.

Constructs containing an ectopic *VSG117* flanked downstream with these mutant 3′UTR sequences were integrated within an active *VSG221* ES in *T. brucei* 221VB1.1 (Sheader *et al*., 2005) (Fig. 6A). Endogenous *VSG221* was subsequently knocked down using tetracycline inducible RNAi to assess the functionality of the ectopic copies of *VSG117*. In most of the cell lines generated, relatively equal amounts of *VSG117* and *VSG221* transcript were expressed. However, *VSG* 3′UTR mutations 2 and 3 significantly affected levels of expression of ectopic *VSG117* (**P < 0.01), and mRNA levels were reduced to 14–16% or 18–20% of wild type respectively (Fig. 6B). The levels of VSG117 protein were also reduced in a comparable fashion (Fig. 6C). These levels of VSG117 expression were not high enough for functional complementation, and cells arrested when endogenous *VSG221* transcript was knocked down with RNAi (Fig. 6D). In some cases, trypanosomes eventually escaped the *VSG* RNAi induced cell cycle arrest (Fig. 6D mut 2), as has been observed previously (Aitcheson *et al*., 2005; Sheader *et al*., 2005). This appears to be a consequence of a negative selection pressure selecting for cells which have escaped the *VSG221* RNAi, either through switching away from *VSG221* or through mutation of a component of the tetracycline inducible RNAi machinery. In contrast, no growth reduction was seen in *T. brucei* expressing *VSG117* flanked by either a wild type *VSG* 3′UTR (navy dotted lines) or other mutant *VSG* 3′UTRs (Supporting Information Fig. S6). Thus, the conserved 16‐mer sequence which was shared between *VSG* 3′UTR mutations 2 and 3, is essential for functional levels of VSG expression.

**Figure 7:**
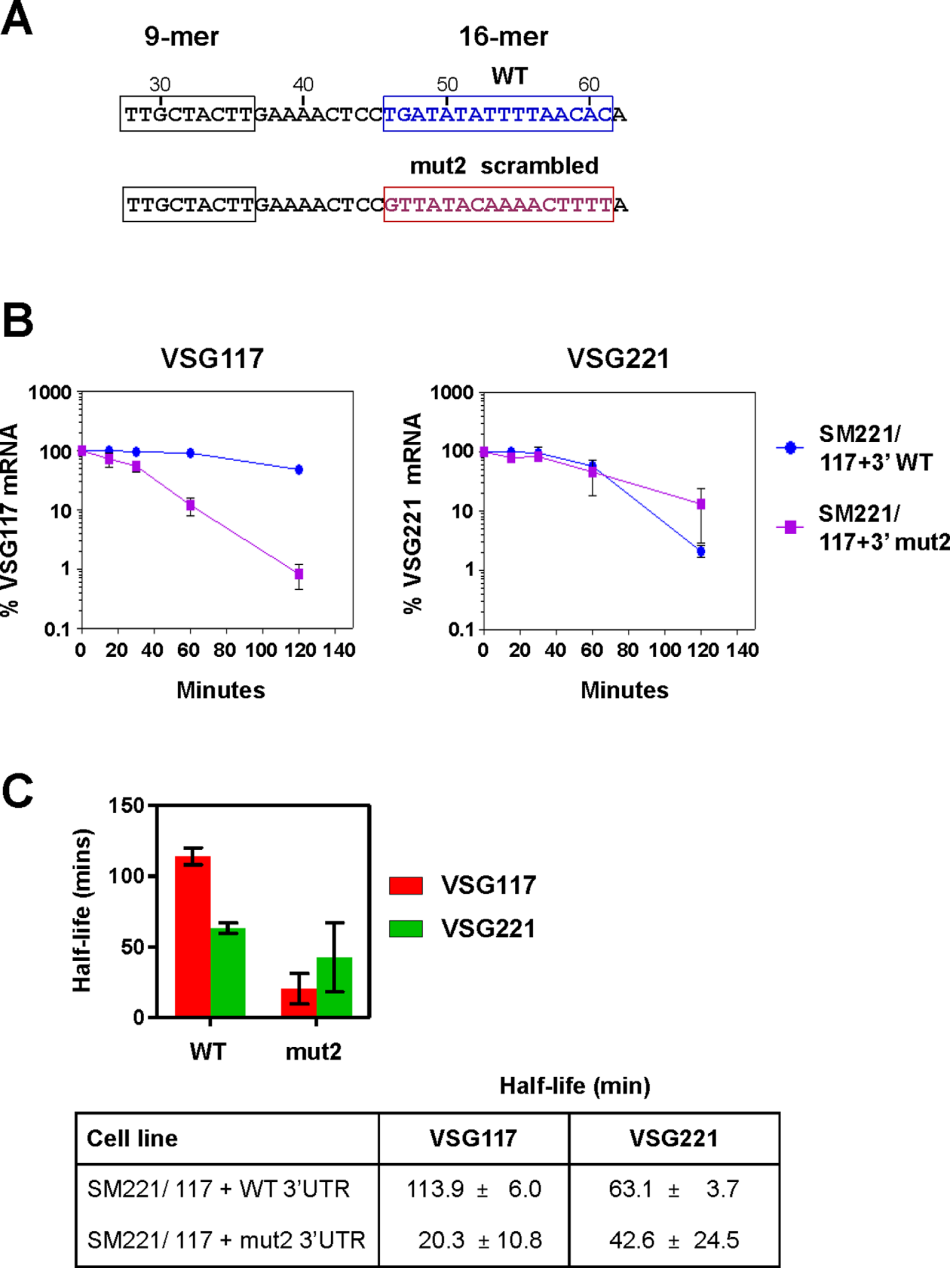
Mutation of a conserved 16‐mer sequence within the *VSG* 3′ UTR results in a drastic reduction in *VSG* transcript half‐life. A. Sequence of a relevant segment of the *VSG117* 3′ UTR with the conserved 9‐mer and 16‐mer sequences highlighted with boxes. The wild type (WT) 16‐mer sequence is shown, as well as the scrambled version present in Mutant 2 (mut 2). Relevant nucleotide numbers are shown in relation to the start of the 3′ UTR. B. Decrease in *VSG* RNA half‐life when the conserved 16‐mer in the *VSG117* 3′UTR is scrambled. RNA was isolated from cell lines expressing *VSG117* with a wild type (WT) *VSG* 3′UTR (*T. brucei* SM221/117 + WT 3′UTR) or a *VSG* 3′ UTR with a scrambled 16‐mer (*T. brucei* SM221/117 + mut2 3′UTR). Cells were incubated with sinefungin to block trans‐splicing and actinomycin D to inhibit transcription, and total RNA was harvested at different time points. Transcript levels were determined by qPCR. Results are presented as a ratio of transcript levels obtained from single‐expresser cell lines expressing either only *VSG117* or *VSG221*. The mean of three independent experiments is shown with the standard deviation indicated with error bars. C. The half‐life in minutes (min) of *VSG117* (red bars) or *VSG221* (green bars) transcript in cell lines where ectopic *VSG117* has either a wild type (WT) or mutant 2 (mut2) 3′UTR. Results are the mean of three independent experiments with the standard deviation indicated with error bars.

We next determined if the conserved 16‐mer in the *VSG* 3′UTR plays a role in transcript stability. This was indeed the case, and scrambling the 16‐mer resulted in a striking reduction in *VSG117* transcript half‐life from 113.9 ± 6.0 min to 20.3 ± 10.8 min (Fig. 7). In contrast, the half‐life of *VSG221* transcript expressed from the endogenous *VSG221* gene was 40–60 min. This value for the *VSG221* mRNA half‐life was significantly lower than the 85–99 min observed earlier, which also corresponds with previously published data (Supporting Information Fig. S2) (Hoek *et al*., 2002). This apparent decrease in *VSG221* mRNA half‐life is presumably due to the presence of a *VSG221* RNAi construct in these cells, which is absent in the cells used for the data presented in Supporting Information Fig. S1. Even in the absence of tetracycline, leaky *VSG221* RNAi presumably degrades *VSG221* mRNA transcript enough to result in an apparent decrease of the *VSG221* half‐life. In support of this explanation, the measured half‐life of *VSG117* mRNA (which would not be affected by *VSG221* RNAi) does not differ drastically between these two different types of cell lines.

Alterations in the polyadenylation site used would affect the length of the 3′UTR, which could potentially impact on mRNA stability (Elkon *et al*., 2013). However, using 3′RACE we did not find a change in the preferred polyadenylation site used for the mRNA of ectopic *VSG117* (Supporting Information Table S3). It is, therefore, possible that an RNA binding protein targeting the conserved 16‐mer in the *VSG* 3′UTR stabilises the *VSG* transcript, although it cannot be excluded that RNA binding proteins binding elsewhere in the transcript are also involved (Clayton, 2014).

### Expression of ectopic VSG117 from the rDNA locus is heterogeneous and does not provide functional levels of expression

**Figure 8:**
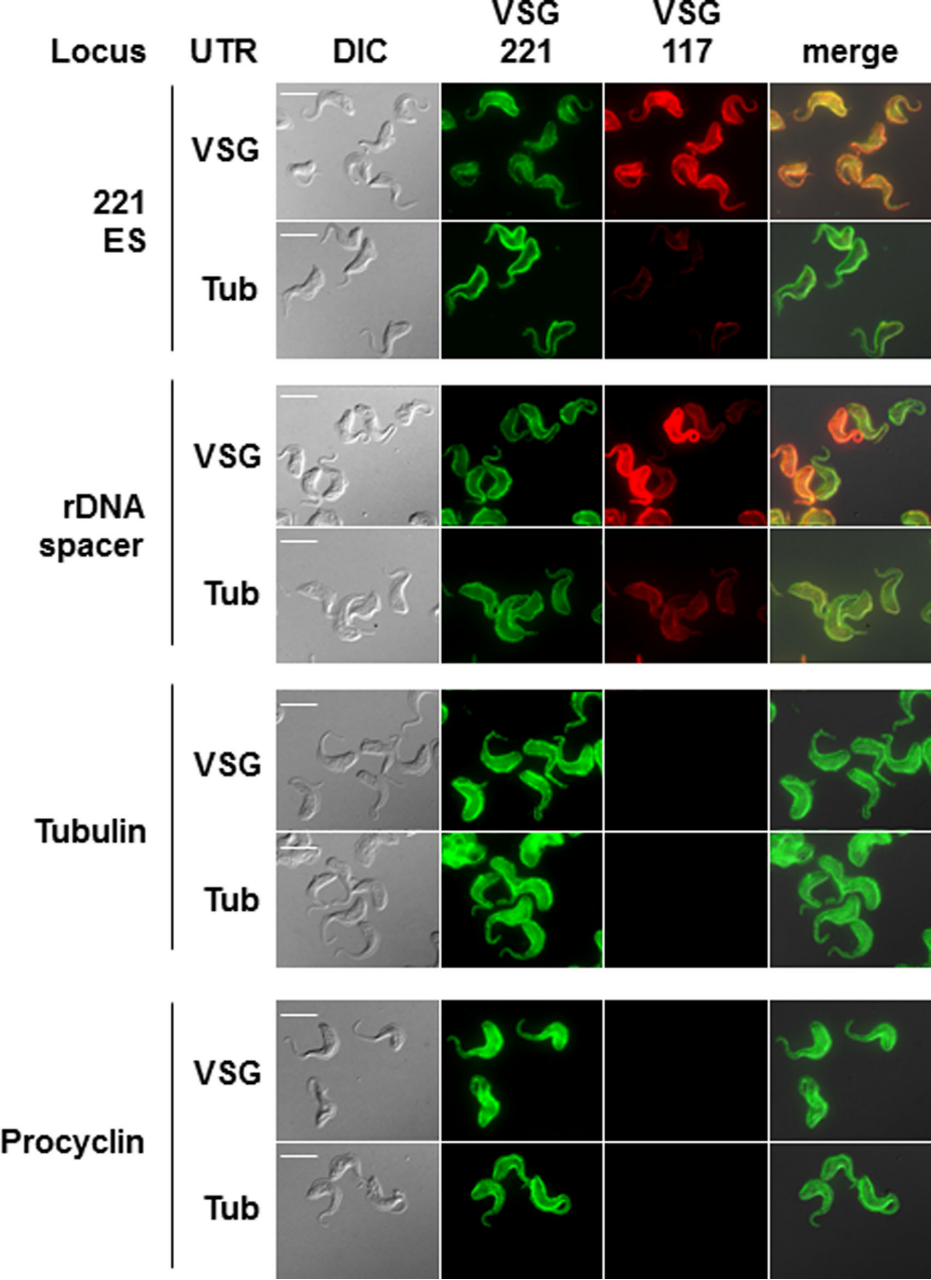
Heterogeneous levels of expression of ectopic *VSG117* expressed from an rDNA spacer compared with an ES. Immunofluorescence analysis was performed on *T. brucei* expressing an ectopic copy of *VSG117* from within the active *VSG221* ES, the rDNA spacer, the tubulin array or upstream of a procyclin locus. The flanking 3′UTR was from *VSG221* or tubulin. Panels show *T. brucei* visualised with differential interference contrast (DIC), or reacted with a rabbit polyclonal antibody specific for VSG221 or a mouse monoclonal against VSG117. The scale bar indicates 10 µM.

Analysis of *T. brucei* expressing ectopic *VSG117* from the active *VSG221* ES showed that both VSG117 as well as VSG221 (encoded by the endogenous *VSG221* gene) were present at the cell surface, and similar homogeneous levels were observed on individual cells (Fig. 8). However, in contrast when *VSG117* was expressed from the rDNA spacer, very heterogeneous levels of VSG117 expression were observed in the population. This variability is presumably a consequence of stochastic changes in the activation state of the rDNA transcription unit.

We, therefore, compared the relative levels of *VSG117* expressed from either the Pol I‐ transcribed ES or the rDNA. If *VSG117* was expressed from the active *VSG221* ES in ‘double‐expresser’ *T. brucei*, *VSG117* transcript levels were approximately 40.4 ± 9.1% those of a ‘single‐expresser’ (Supporting Information Fig. S7A). Similarly, at the population level transcript levels from a gene inserted in the rDNA spacer were approximately 31.4 ± 5.6% of a ‘single‐expresser’. Similarly at the protein level, VSG117 expressed from the active ES was 54.1 ± 6.1% ‘single‐expresser’ levels, while there was marginally reduced expression from the rDNA spacer (41.9 ± 17.3% levels of a VSG117 ‘single‐expresser’) (Supporting Information Figs S6B and S6C). Both the SM221/117 and 221rD117 cell lines include *VSG221* RNAi constructs. However, in the absence of tetracycline, we do not think that leaky expression from these constructs significantly impacts on our measurements. The expression of ectopic *VSG117* appears to increase after knock‐down of *VSG221* in the SM221/117 cells, but not in the 221rD117 cell‐line. However, this is presumably a consequence of the SM221/117 (but not 221rD117) cells continuing to proliferate, and thereby skewing the population towards VSG117 expressers.

### A precise cell cycle arrest is not triggered in cells stalled by the expression of ectopic VSG from the rDNA spacer

**Figure 9:**
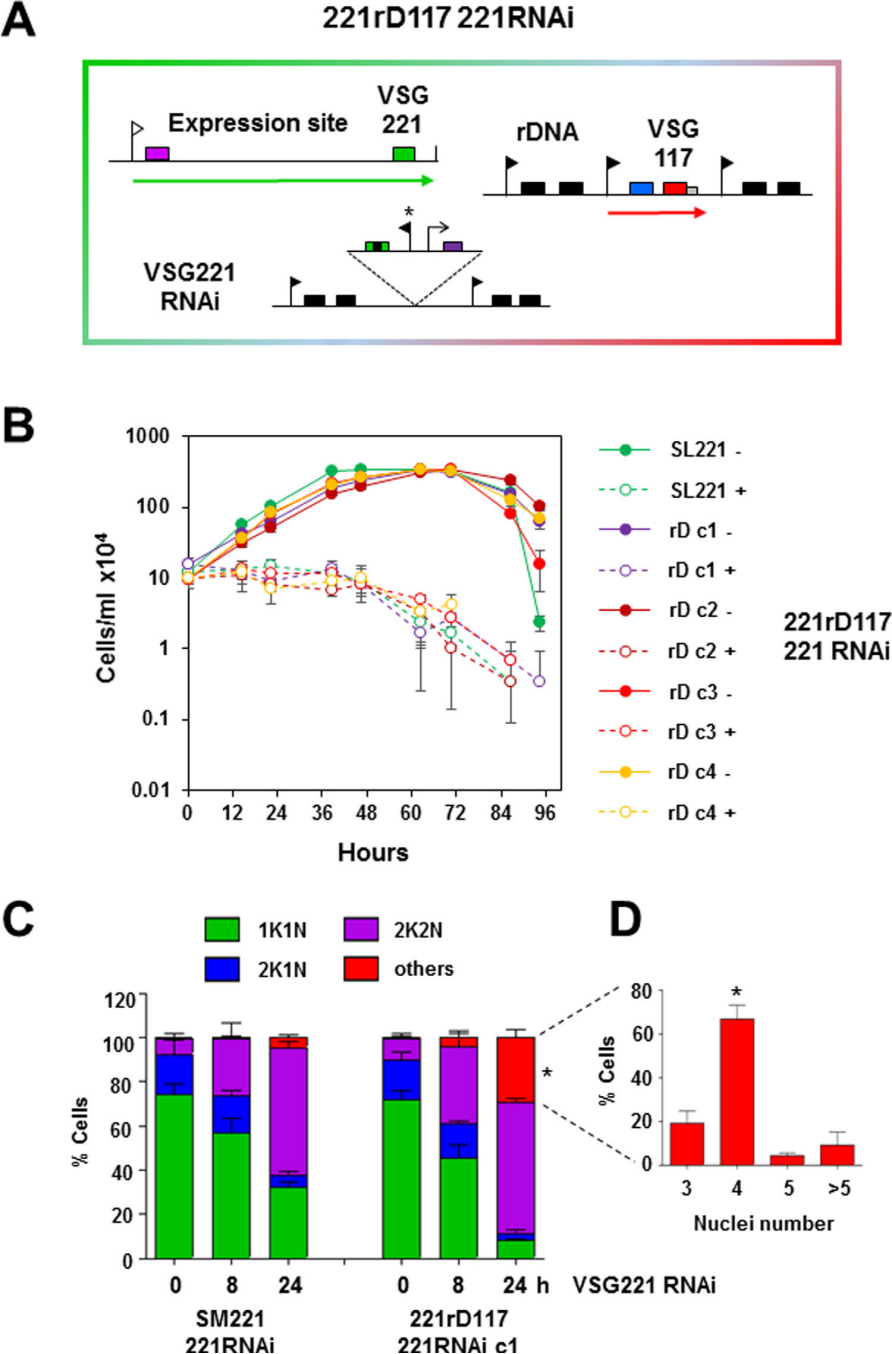
VSG expressed from the rDNA spacer does not functionally complement the cell and results in cells arresting precytokinesis without triggering a characteristic ‘VSG synthesis block’ cell‐cycle checkpoint. A. Schematic of the *T. brucei* 221rD117 221RNAi cell line. Transcription of the active *VSG221* ES is selected for using a puromycin resistance gene (purple box). A construct containing *VSG117* (red box) and a hygromycin resistance gene (blue box) is integrated in a tagged rDNA spacer. *VSG221* RNAi was induced using a tetracycline inducible RNAi stem‐loop construct inserted in the rDNA (indicated with black boxes). The ES promoter is indicated with a white flag, the rDNA promoter with black flags, the tetracycline inducible promoter with an arrow, and the tetracycline inducible rDNA promoter indicated with an asterisk. B. Growth curves in the presence (+) or absence (‐) of tetracycline induced *VSG221* RNAi. The SL221 cell line that does not contain ectopic *VSG117*. Four *T. brucei* 221 rD117 221 RNAi clones expressing ectopic *VSG117* from the rDNA are compared (rD c1‐c4). The cell density over time was monitored. Results are from three biological replicates with the standard deviation indicated with error bars. C. Cell karyotype was determined using microscopy with cells where the DNA was stained with DAPI. The numbers of kinetoplasts (K) or nuclei (N) were determined with the expanded section detailing the karyotype of cells described as ‘others’. The number of cells described as ‘others’ was significantly increased (*P < 0.05, unpaired *t* test) when *VSG221* RNAi was induced in cells where ectopic *VSG117* was expressed from the rDNA spacer (221rD117 221RNAi) compared to when there is no ectopic *VSG* expressed. Standard deviation from three biological replicates is indicated with error bars (*n* ∼ 200 was counted for each time point in all biological replicates). D. Within the karyotype category of ‘others’, there was a significant predominance of cells with 4 nuclei, indicating that they had undergone an additional round of re‐initiation of S phase (*P < 0.05, ANOVA followed by Tukey *post hoc*).

We next asked if VSG117 expressed from the rDNA spacer could functionally complement the cell if VSG221 synthesis was knocked down using RNAi (Fig. 9A). In the presence of *VSG221* RNAi all *T. brucei* clones investigated stalled abruptly, indicating that expression of ectopic *VSG117* from the rDNA spacer was not adequate for survival (Fig. 9B). We next performed cell cycle analysis on cells that had stalled after *VSG221* synthesis was blocked in the presence of expression of ectopic *VSG117* from the rDNA (Fig. 9C). Cells in G1 have one kinetoplast (K) and one nucleus (N). As they enter S phase, first the kinetoplast divides (2K1N), and then mitosis occurs generating (2K2N) cells. In the parental *T. brucei* SM221 221RNAi cell line, there was an expected accumulation of 2K2N cells after the induction of *VSG221* RNAi from 7.3 ± 2.1% to 57.7 ± 2.6% (Sheader *et al*., 2005). If *VSG221* RNAi was induced in the presence of ectopic *VSG117* expressed from the rDNA spacer, an increase in 2K2N cells was also observed (9.9 ± 2.3% to 34.7 ± 7.3%). However, strikingly, there was also an increase in multi‐nucleated cells (‘others’) (0.3 ± 0.6% to 29.2 ± 3.4%) (*P < 0.05) (Fig. 9D). Most of these multi‐nucleated cells had four nuclei (66.9 ± 6.1%) (*P < 0.05) indicating that they had stalled prior to cytokinesis, but had re‐entered S‐phase. Therefore, although VSG expression levels from the rDNA were not consistently at high enough levels to allow cytokinesis, they were also not consistently low enough to trigger the precise ‘VSG synthesis block’ cell‐cycle checkpoint. There were relatively few cells with more than four nuclei. Possibly the concurrent (and previously documented) global translation arrest prevented cells from reinitiating S phase more than once (Smith *et al*., 2009).

## Discussion

Extraordinarily high levels of VSG are expressed from a single active *VSG* gene in bloodstream form *T. brucei*. Here we investigate the role of different genomic features facilitating this. We show that functional levels of VSG could be expressed from an ectopic *VSG* gene located more than 60 kb upstream of its normal telomeric location within the active ES. A *VSG* 3′UTR was key for high VSG expression levels, and a conserved 16‐mer sequence was essential for stabilising the *VSG* transcript. High levels of VSG expression were only obtained from Pol I‐transcribed loci including the ES and the rDNA. However, these two different Pol I transcription units were not comparable. Expression of ectopic *VSG* from the rDNA spacer was highly heterogeneous at the population level, presumably as a consequence of variability in the activation state of the ectopic rDNA promoter and surrounding rDNA transcription units. VSG expressed from the rDNA spacer did not functionally complement the cell. The cells arrested prior to cytokinesis, as was also observed after blocking VSG synthesis. However, many cells re‐entered S‐phase, indicating that VSG levels were not consistently low enough to trigger the precise ‘VSG synthesis block’ cell cycle checkpoint in all cells.

There is approximately 700‐fold more *VSG* mRNA than *ESAG1* mRNA generated in bloodstream form *T. brucei*, even though these two different genes are transcribed from the same polycistronic ES transcription unit (Cully *et al*., 1985). The *VSG* 3′UTR has previously been shown to play a role in stabilising transcript from a CAT reporter gene three‐fold compared to CAT without a flanking 3′UTR in bloodstream form *T. brucei* (Berberof *et al*., 1995). These experiments also showed that introducing a *VSG* 3′UTR downstream of the CAT reporter gene resulted in its developmental regulation. Here, we also show that the *VSG* 3′UTR is essential for the production of functional levels of *VSG* transcript, and show essentiality of the conserved *VSG* 3′UTR 16‐mer sequence, which when scrambled resulted in a dramatic reduction in *VSG* transcript half‐life from 113.9 ± 6.0 to 20.3 ± 10.8 min.

Transcriptomic analyses comparing mRNA stability at a whole genome level have shown that the median half‐life of mRNA transcripts in bloodstream *T. brucei* is 13 min (Manful *et al*., 2011; Clayton, 2014; Fadda *et al*., 2014). The *VSG* transcript has an unusually long half‐life. It is possible that this is due to a protein binding the conserved 16‐mer in the *VSG* 3′UTR. On disruption of this putative interaction, *VSG* transcript stability was reduced down to a level which is average for mRNA in bloodstream form *T. brucei*. This hypothetical 16‐mer binding factor could act directly to protect the mRNA transcript from degradation by physically blocking association with a nuclease. Alternatively these 16‐mer sequences, or a protein binding them could promote translation. There is evidence that translation is a major factor in preventing mRNA decay in *T. brucei*, and inhibition of translation with different inhibitors including cycloheximide and puromycin significantly reduces the half‐life of *VSG* mRNA (Ehlers *et al*., 1987; Delhi *et al*., 2011). Using *in vitro* methods, we have not yet been successful in identifying a hypothetical RNA binding protein.

Mutation of the conserved 9‐mer sequence did not have an observable effect on transcript functionality. However, the *VSG* 3′UTR is not only important for transcript stability, but also plays a role in DNA recombination. During a chronic infection, new *VSG* variants are frequently copied into the active ES via gene conversion (Vink *et al*., 2012; McCulloch *et al*., 2015). Upstream homology is provided by characteristic 70 bp repeats, and downstream homology by conserved regions in the *VSG* 3′UTR (Hovel‐Miner *et al*., 2016). In addition to affecting transcript stability, these 3′UTR sequences including the conserved 9‐mer could, therefore, play an additional role in facilitating gene conversions.

We show that functional VSG levels could be obtained from an ES promoter proximal *VSG* gene located 60 kb upstream of its normal location at the chromosome end. However, in wild‐type *T. brucei* the active *VSG* is invariably the last gene within the ES, and adjacent to the telomere repeats. One explanation for this highly conserved genomic architecture of the ES, is that the telomere proximal location of *VSG* could facilitate *VSG* silencing in inactive ESs. There is a low level of leaky transcription from ‘inactive’ ES promoters, which attenuates within the ES (Vanhamme *et al*., 2000; Kassem *et al*., 2014). The chromatin protein TbRAP1 mediates a repressive silencing gradient operating on inactive ESs, which is stronger immediately near the telomere end, compared with 60 kb upstream (Yang *et al*., 2009; Nanavaty *et al*., 2017). Efficient suppression of transcription of *VSG*s located in silent ESs is essential for the mono‐allelic expression of the active *VSG* (Duraisingh and Horn, 2016). The region immediately proximal to the telomere repeats within the inactive ES could, therefore, be the most transcriptionally silent location within the ES to locate *VSG*.

In addition, the invariably telomeric location of the active *VSG* would facilitate the DNA rearrangements mediating *VSG* switching. DNA recombination reactions at telomeres mediate the generation of sequence diversity within polymorphic gene families involved in phenotypic or antigenic variation which are frequently telomeric (Keely *et al*., 2005; Scherf *et al*., 2008; de Las Penas *et al*., 2015; Lue and Yu, 2017). In *T. brucei* this is also the case, and the telomeric *VSG* can be switched either through telomere exchange or through gene conversions (Li, 2015; McCulloch *et al*., 2015). Subtelomeres in *T. brucei* are particularly fragile regions of the genome, and are susceptible to DNA strand breaks facilitating *VSG* switching (Glover *et al*., 2013a; Nanavaty *et al*., 2017). An important reason that *VSGs* are invariably located at the ends of the ES transcription units, is presumably because this is the most recombinogenic place for them to be (Hovel‐Miner *et al*., 2012).

High levels of ectopic *VSG* could only be expressed from Pol I transcription units including the ES and the rDNA, compared with the low levels obtained from the Pol II‐ transcribed αβ‐tubulin transcription unit. Pol I transcription units are unique in being transcribed at an extremely high rate, facilitated by both high rates of RNA polymerase initiation, and density of polymerase loading on the DNA template (French *et al*., 2003; Viktorovskaya and Schneider, 2015). In *T. brucei* this is also the case, and it has been estimated that a marker inserted in a Pol I transcription unit is transcribed at a 10‐fold higher rate than one inserted in a Pol II transcription unit (Biebinger *et al*., 1996). Pol I‐derived transcripts are uncapped, and, therefore, untranslateable (Grummt and Skinner, 1985). However, as *T. brucei* adds a capped Pol II‐derived spliced leader RNA to the 5′ ends of its mRNAs through trans‐splicing, it can use Pol I to direct expression of protein coding genes (Gunzl, 2010). *T. brucei*, therefore, appears to have recruited its strongest (Pol I) promoter to direct transcription of *VSG*.

In our experiments, expression of ectopic *VSG117* from a variety of genomic locations appeared to lead to a decrease in levels of the endogenous *VSG221* transcript, with the relative levels of the two *VSGs* approximately inversely correlated with each other. In previous experiments by Batram *et al*, expression of a second ectopic *VSG* from a tetracycline inducible T7 promoter resulted in down‐regulation of the endogenous telomeric *VSG* through chromatin mediated silencing of the active ES telomere (Batram *et al*., 2014). In contrast, we did not see a statistically significant decrease in transcription of the active *VSG221* ES telomere after expression of ectopic *VSG117* from either the ES or the rDNA spacer.

The discrepancy of our results with those of Batram *et al* is presumably a consequence of exactly how the ectopic *VSG* is expressed. Batram *et al*. investigate a transient phenomenon which occurs within an 8 h period, as the induction of the T7 promoter rapidly directs large amounts of transcription of the ectopic *VSG*. This resulted in the generation of stumpy forms, possibly as a consequence of a stress response (Batram *et al*., 2014; Zimmermann *et al*., 2017). It has been shown that as *T. brucei* differentiates to the stumpy form, transcription of the active *VSG* is silenced through transcription attenuation progressing upwards along the ES from the chromosome end (Amiguet‐Vercher *et al*., 2004). It is, therefore, possible that the transcriptional attenuation of the active ES observed by Batram *et al*. is a result of the cell becoming ‘stumpy‐like’, rather than a direct consequence of expression of the ectopic *VSG* as such. In this inducible system, expression of the ectopic *VSG* is silenced within a number of days through an unknown mechanism, and expression of the endogenous *VSG* returns to normal. In contrast in our experiments, we investigated stable transformants which have adjusted to the expression of a second ectopic *VSG*. As the amount of endogenous *VSG221* transcript was consistently in approximately inverse proportion to the amount of ectopic *VSG* expressed, our results are best explained by the presence of a factor binding the *VSG* 3′UTR. If this protein is not in excess, it would restrict the total amount of *VSG* transcript which can be stably expressed in the cell.

Although *VSG* ESs and rDNA transcription units are both transcribed by Pol I, they show very different properties. Expression of ectopic *VSG117* located within the active ES resulted in homogeneous expression of VSG117. In contrast, expression of ectopic *VSG117* from a specific tagged rDNA promoter within the Pol I‐transcribed rDNA spacer resulted in highly heterogeneous VSG117 expression at the population level. It has been shown earlier that not all rDNA loci in *T. brucei* are equivalent, and that varying levels of expression can be generated from constructs integrated into different rDNA spacers (Alsford *et al*., 2005). To eliminate complications due to this putative position effect, we have performed all of our analyses using a single tagged rDNA locus. In addition to possible position effects, it has earlier been shown that ectopic expression of proteins from an rDNA promoter was reduced at high cell culture densities in bloodstream form *T. brucei* (Ali and Field, 2013). To eliminate this complication, we have performed all of our experiments using mid‐logarithmic stage parasites at approximately equivalent cell densities.

Our data showing heterogeneous levels of expression from a single tagged rDNA locus is, therefore, presumably due to alternating activation states of the *T. brucei* rDNA promoter. It has been shown that rDNA transcription units in *Saccharomyces cerevisiae* and mammalian cells exist in different fluctuating activation states. Within an individual cell, typically about half of the rDNA transcription units are transcriptionally active, while the other half are silenced at the level of chromatin (McStay and Grummt, 2008; Grummt and Langst, 2013; Hamperl *et al*., 2013). This is presumably also the case in *T. brucei*. This very different transcriptional behaviour between the ES and rDNA highlights key differences in these Pol I transcription units.

The inability of *VSG* expressed from an rDNA promoter to functionally complement the cell is presumably due to fluctuating levels of activity of the rDNA promoter. However, these stalled cells did not universally trigger a ‘VSG synthesis block’ cell cycle checkpoint (Sheader *et al*., 2005). Although there was a large accumulation of post‐mitotic 2K2N cells there was also a significant increase in multi‐nucleated cells, indicating that they had reinitiated S‐phase. This argues that the ‘VSG synthesis block’ checkpoint is a stress response, which is the consequence of consistent depletion of VSG in all cells. The pulsating levels of VSG expressed from the rDNA promoter possibly allowed more cells to proceed through G1 (1K1N) to a precytokinesis stage (2K2N), after which some cells then reinitiate S‐phase. It will be interesting to determine exactly how much continuous depletion of VSG is required to trigger this unique cell‐cycle checkpoint, and how VSG levels are likely being sensed.

In summary, we identify key features required for the expression of functional levels of VSG in bloodstream form *T. brucei*. Not only must the active *VSG* be located within a Pol I‐transcribed ES, but it needs to be flanked by a *VSG* 3′UTR with a conserved 16‐mer sequence. Our experiments also highlight critical differences between the ES and rDNA Pol I transcription units. Although very high levels of expression can be generated from rDNA promoters, these levels fluctuate, and are not sufficient to generate functional levels of VSG expression. These results highlight the molecular adaptations that the bloodstream form trypanosome has adopted to ensure that 10% of its protein can be produced from a single Pol I‐transcribed *VSG* gene. They also highlight the essentiality of close to normal levels of VSG expression for the viability of bloodstream form *T. brucei*, allowing it to be an effective pathogen of the mammalian bloodstream.

## Experimental procedures

### Trypanosome strains and culturing

Bloodstream form *Trypanosoma brucei brucei* 427 was used for all experiments and was cultured in HMI‐9 medium with 15% foetal calf serum. All cell lines used in this study are detailed in Supporting Information Table S4. Many are based on the *T. brucei* ‘single marker’ SM cell line (Wirtz *et al*., 1999) or a derivative with a puromycin resistance gene (pur) incorporated immediately behind the promoter of the active *VSG221* ES (SM221) (Narayanan *et al*., 2011; Stanne *et al*., 2011) where it is called S16.221. The presence of a selectable marker in the active *VSG* ES prevents switching to another *VSG* ES. The *T. brucei* cell line expressing both VSG117 and VSG221 has an ectopic copy of *VSG117* inserted immediately behind the promoter of the active *VSG221* ES. Here *VSG117* has an α‐tubulin splice site and a *VSG221* downstream region containing the *VSG221* 3′UTR and polyadenylation signal (Smith *et al*., 2009). To generate a cell line dependent on VSG117 expressed from the *VSG221* ES, the subtelomeric *VSG221* gene in *T. brucei* SM221/117 was exchanged with a blasticidin resistance gene using the pBSVSG221KOblast construct to generate *T. brucei* SMΔ221/117. Otherwise, all other constructs integrated in the *VSG221* ES in this study were targeted 216 bp downstream of the transcription start site.

To study the effect of *VSG117* expression from different *T. brucei* genomic loci, constructs containing a hygromycin resistance gene and *VSG117* were integrated into different genomic regions in the *T. brucei* SM221pur cell line. In these constructs, *VSG117* was flanked upstream by aldolase RNA processing sequences, and downstream by either the *VSG221* downstream region (containing the 3′UTR and polyadenylation signal) or the intergenic region downstream of an α‐tubulin gene. These ectopic copies of *VSG117* were targeted to the active *VSG221* ES (*T. brucei* SM221/117VSG3′UTR and SM221/117Tub3′UTR), the α‐β tubulin array (SM221/tub117VSG3′UTR and SM221/tub117Tub3′UTR), and upstream of the PARP B1 procyclin locus (SM221/pro117VSG3′UTR and SM221/pro117Tub3′UTR) (Rudenko *et al*., 1990). To integrate *VSG117* into a specific rDNA spacer, a ‘landing‐pad’ construct (prDNATargrProeGFPBSD) containing *eGFP* and a blasticidin resistance gene was first used to create the *T. brucei* SM221/rDNAeGFP cell line. Subsequently, this cell line was used to integrate *VSG117* into this marked rDNA spacer using the same rDNA targeting sequence and part of the blasticidin gene for homology (SM221/rDNA117VSG3′UTR and SM221/rDNA117Tub3′UTR). In constructs used to integrate *VSG117* into either the procyclin locus or the rDNA spacer, an rDNA promoter in the construct directed transcription of the drug resistance gene and *VSG117*.

A construct containing *VSG117* with downstream chimeric 3′ sequences was integrated into the active *VSG221* ES of the *T. brucei* SM221pur cell line (SM221/117 VSG‐Tub3′and SM221/117 Tub‐VSG3′). In VSG‐Tub, the chimeric 3′ sequences are composed of the *VSG221* 3′UTR (76 bp) flanked by sequences immediately downstream of the α‐tubulin 3′UTR (187 bp). In Tub‐VSG the α‐tubulin 3′UTR (92 bp) is flanked by *VSG221* sequences immediately downstream of the *VSG221* 3′UTR (538 bp).

Six *VSG117* 3′UTR mutants were generated to analyse the *VSG* 3′UTR in more detail. Details of the mutations are in shown in Fig. 5 and Supporting Information Table S2. Constructs contained a puromycin resistance gene and a *VSG117* gene preceded by an α‐β tubulin intergenic region and flanked downstream by a *VSG117* 3′UTR which was either wild type or mutant. These were integrated into *T. brucei* 221VB1.1 (Sheader *et al*., 2005), thereby replacing the blasticidin resistance cassette present in the *VSG221* ES. This cell line contains a *VSG221* RNAi construct with opposing tetracycline inducible T7 promoters. The *T. brucei* SM221/117 221RNAi cell line is referred to as *T. brucei* 221VP117 in (Smith *et al*., 2009). *T. brucei* 221rD117 221RNAi is *T. brucei* SM221/rDNA117VSG3′UTR with a stem‐loop *VSG221* RNAi construct generated from pLEW100v5xPEX11 (Silverman *et al*., 2011). The *T. brucei* SL221 cell line is *T. brucei* SM221pur transfected with this stem‐loop *VSG221* RNAi construct. The VSGV02 expressing cell line *T. brucei* HNI(V02)(Rudenko *et al*., 1998) was also used in Western blot and qPCR analyses.

### DNA constructs

The DNA constructs used in this study are detailed in Supporting Information Table S4. Primers used for cloning are shown in Supporting Information Table S5. Additional sequences and cloning details are available on request. The construct pBSVSG221KOblast contains a blasticidin resistance gene between *VSG221* upstream (706 bp) and downstream (602 bp) sequences which provided RNA processing signals and served as targeting fragments. Sequences which were used to target constructs immediately downstream of the *VSG221* ES promoter can be found in (Sheader *et al*., 2004). In constructs where ectopic *VSG117* was integrated into different genomic locations, the hygromycin gene is flanked upstream by a tubulin splice acceptor sequence and downstream by an aldolase intergenic region containing polyadenylation sequences and a 5′ splice acceptor site. The *VSG117* gene was flanked downstream by *VSG221* 3′ sequences including a 3′UTR and polyadenylation sequences. In addition, analogous constructs had *VSG117* flanked with the 3′ downstream region from α‐tubulin. These ectopic copies of *VSG117* were inserted into various genomic loci. These include immediately downstream of the *VSG221* ES promoter, within a specific rDNA spacer, within the constitutively transcribed tubulin array (α and β tubulin genes indicated with grey boxes) or upstream of the silent procyclin transcription units. To integrate *VSG117* into a tagged rDNA spacer selected for high expression, the same rDNA upstream targeting was used as in prDNA Targ rPro eGFP BSD.

To generate the VSG‐Tub chimeric 3′ sequences, the *VSG221* 3′UTR and α‐tubulin downstream sequences were amplified from the p221_117 + VSG3′UTR and p221_117 + Tub3′UTR constructs respectively using a long primer which covers the join between the 3′UTR and the downstream sequences. Subsequently these PCR products were used as the template in a third PCR reaction with a primer annealing to the start of the *VSG* 3′UTR and a primer annealing to the end of the α‐tubulin downstream sequence. A similar process was followed to produce the Tub‐VSG chimeric 3′sequences. To generate the *VSG* 3′UTR mutant constructs, a plasmid containing *VSG117* flanked downstream by the *VSG221* 3′UTR and downstream sequences was digested with PstI and XbaI to exchange part of the *VSG117* ORF and the *VSG221* 3′UTR with 277 bp of synthesised DNA (either wild type or mutant)(GenScript). The *VSG117* UTR was flanked downstream with *VSG221* polyadenylation sequences, as *VSG117* polyadenylation sequences are not available in our *T. brucei* cell line. The entire *VSG117* ORF with *VSG117* 3′UTR and *VSG221* downstream sequences was then cloned into p221purVSG117UTR (Smith *et al*., 2009) to create the 3′UTR mutant series of constructs. The stem loop RNAi construct targeting *VSG221* was generated from pLEWv5xPEX11 (Silverman *et al*., 2011).

### Western blot analysis

For chemiluminescent experiments, cells were washed once at 4°C, and lysed in protein lysis buffer (50 mM Hepes pH 7.5, 10% glycerol, 1% Triton X‐100, 1.5 mM MgCl_2_, 1 mM EGTA, a protease inhibitor tablet (Roche), and incubated at 4°C for 20 minutes on a rotating wheel. Prior to SDS‐PAGE, the protein lysates were combined with 4x protein loading buffer and boiled for 5–10 minutes. Protein lysates were electrophoresed on a 10% resolving gel and transferred onto a Hybond‐P membrane (GE Healthcare). The membrane was probed with rabbit polyclonal antibodies against VSG221, VSG117 or BiP. Protein was visualised using anti‐rabbit ECL peroxidase (GE Healthcare) and Super Signal West Pico chemiluminescent substrate (Thermo Scientific).

In the Li‐Cor Western blotting experiments cells were lysed in 50 mM HEPES (pH 7.5); 1 mM EGTA; 1.5 mM MgCl_2_; 10% (v/v) glycerol, 1% (v/v) Triton X‐100 with a cocktail of protease inhibitors (2 ug/ml of leupeptin, chymostatin, pepstatin and antipain). Protein lysate was suspended in loading buffer, and boiled at 100°C for 10 min before loading. The equivalent of 2 × 10^4^ cells per well was electrophoresed and transferred onto Hybond‐P membrane. Blots were probed in Odyssey Blocking buffer (Li‐Cor) and washed with 0.1% PBS‐Tween between probes. Blots were probed with the rabbit primary antibodies (from Jay Bangs): anti‐VSG221, anti‐VSG117 and anti‐BiP and the mouse KMX‐1 antibody (from Keith Gull). Secondary antibodies used were: IRDye 680LT anti‐rabbit IgG (H + L) and IRDye 800CW anti‐mouse IgG (H + L) (both from Li‐Cor). Protein bands were visualised and quantified using the Odyssey® Infrared Imaging System.

### Immunofluorescence microscopy

For immunofluorescence microscopy experiments, cells were washed twice in cold PSG buffer and fixed with 2% paraformaldehyde at room temperature for 15 min. Cells were washed twice in PBS buffer and allowed to settle on ColorFrost Plus microscopy slides (Shandon) for 30 min in a humidity chamber. The slides were then washed with PBS buffer and probed simultaneously with either rabbit polyclonal anti‐VSG221 or mouse monoclonal anti‐VSG117 (both kind gifts from Jay Bangs) followed by AlexaFluor 488 conjugated goat anti‐rabbit (Invitrogen) and Dylight 594 conjugated goat anti‐mouse (Thermo). All probing steps were carried out for 45 min in a humidity chamber and followed by a wash with PBS buffer. The slides were subsequently mounted in Vectashield with DAPI (Vector Laboratories). Cells were imaged with an M1 Imager fluorescence microscope (Zeiss) using an AxioCam MRm camera. Post‐acquisition analyses were carried out with ImageJ (National Institutes of Health, Bethesda, MD, USA).

### RNA analysis

Total RNA was isolated using a Qiagen RNeasy Mini Kit (Qiagen) and genomic DNA was removed using TurboDNAse (Ambion), and DNase removed using DNAse Inactivation Reagent (Ambion) according to the manufacturer's instructions. Synthesis of cDNA was carried out with 100 ng RNA per sample using an Omniscript RT kit (Qiagen). qPCR reactions were performed using Brilliant II SYBR low ROX master mix (Agilent) with relevant primers (Supporting Information Table S6) using an Applied Biosystems 7500 real time PCR machine. qPCR reactions were performed with 1 µl cDNA diluted ten‐fold with the exception of the mRNA decay assays where the cDNA was diluted 100‐fold. Relative quantification of mRNA was performed with each sample normalised to levels of actin transcript. Unless stated otherwise, qPCR data are presented as arbitrary units (2^‐ΔCt^). Primers used for qPCR to detect *VSG221* transcript amplify a region of the *VSG221* gene located at 1058–1184 bp, and downstream of the region used for *VSG221* RNAi (106–910 bp). Predicted RNA secondary structure was determined using RNAfold (University of Vienna) (ViennaRNA Package 2.0) (Gruber *et al*., 2008).

### RNA stability and polyadenylation site analysis

For the mRNA stability assays, bloodstream form *T. brucei* was grown to midlog phase (1 × 10^6^ cells ml^−1^). Cells were incubated with Sinefungin (Millipore) at 5 µg ml^−1^ for 5 min at 37°C to inhibit trans‐splicing and Actinomycin D (10 µg ml^−1^) was added to block transcription. Cells were harvested at various time points, RNA was isolated and cDNA generated and quantified as detailed above. For the mRNA stability experiments, *VSG* mRNA was quantified in comparison with 28Sβ rRNA rather than actin. Mean values were calculated with data from three independent experiments. Primer sequences used for qPCR are shown in Supporting Information Table S6.

Analysis of RNA polyadenylation sites was carried out using 3′ RACE (Rapid amplification of cDNA ends) based on methods described in (Scotto‐Lavino *et al*., 2006) with minor variations. Briefly RNA (1 µg) was incubated at 80°C for 3 min, then placed on ice. cDNA was generated using Omniscript (Qiagen) and the Q_T_ primer (50 ng) in a 20 µl reaction mix. Samples were incubated for 5 min at room temperature, 42°C for 1 h, 50°C for 10 min, then 70°C for 15 min. At the end of the reaction 1 ml TE buffer was added. PCR was performed as described in (Scotto‐Lavino *et al*., 2006) using the Q_0_ RACE primer and the *VSG117* specific RACE primer for the first round and the Q_1_ RACE primer paired with *VSG117* primer 2 for the second. PCR products were cloned into pCR™4Blunt‐TOPO® and sequenced. The sequences of all RACE primers are in Supporting Information Table S6.

### Statistical analyses

Statistical analyses of transcript and precursor transcript levels using qPCR were carried out using GraphPad Prism 5.0. One‐way ANOVA was carried out for each experimental set to determine overall significance of difference followed by Tukey *post hoc*. Significance of difference for Li‐Cor Western blots was carried out using pairwise *t* tests. For all data analyses, differences were considered significant with *P* ≤ 0.05.

## Supporting information

Supporting Figures and Tables
