# Supplementary material for: The role of genomic location and flanking 3′UTR in the generation of functional levels of variant surface glycoprotein in Trypanosoma brucei
Source: Mol Microbiol. 2017 Oct 11;106(4):614–34. doi: 10.1111/mmi.13838 (PMC5698767; doi:10.1111/mmi.13838)
Supplement: Supplementary file 1 — Supporting Figures and Tables [file MMI-106-614-s001.pdf]

Supplemental Information

For:

The role of genomic location and flanking 3'UTR in the generation of functional levels of Variant Surface Glycoprotein in Trypanosoma brucei

By:

Sophie Ridewood, Cher-Pheng Ooi, Belinda Hall, Anna Trenaman, Nadina Vasileva Wand, Georgios Sioutas, Iris Scherwitzl and Gloria Rudenko

Containing:

7 Figures and figure legends
and
6 Tables

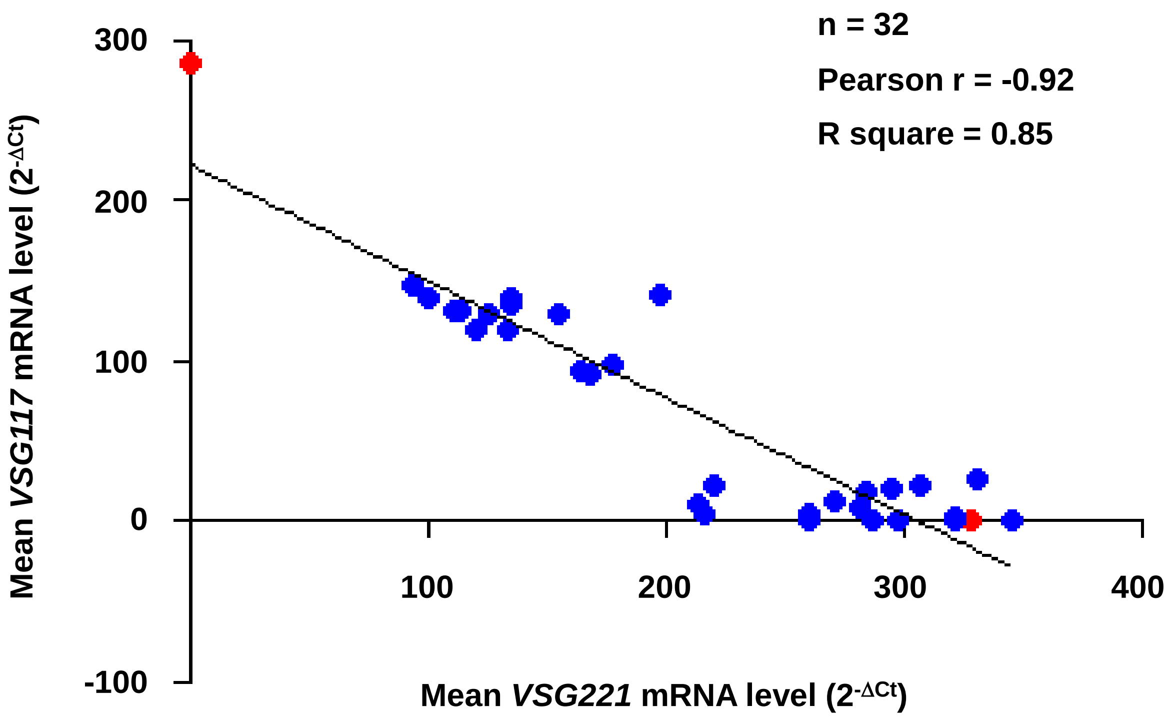
n = 32
Pearson r = -0.92
R square = 0.85
300
200
100
0
-100
100
200
300
400
Mean VSG117 mRNA level ($2^{-\Delta Ct}$)
Mean VSG221 mRNA level ($2^{-\Delta Ct}$)

Sup Fig S1

A

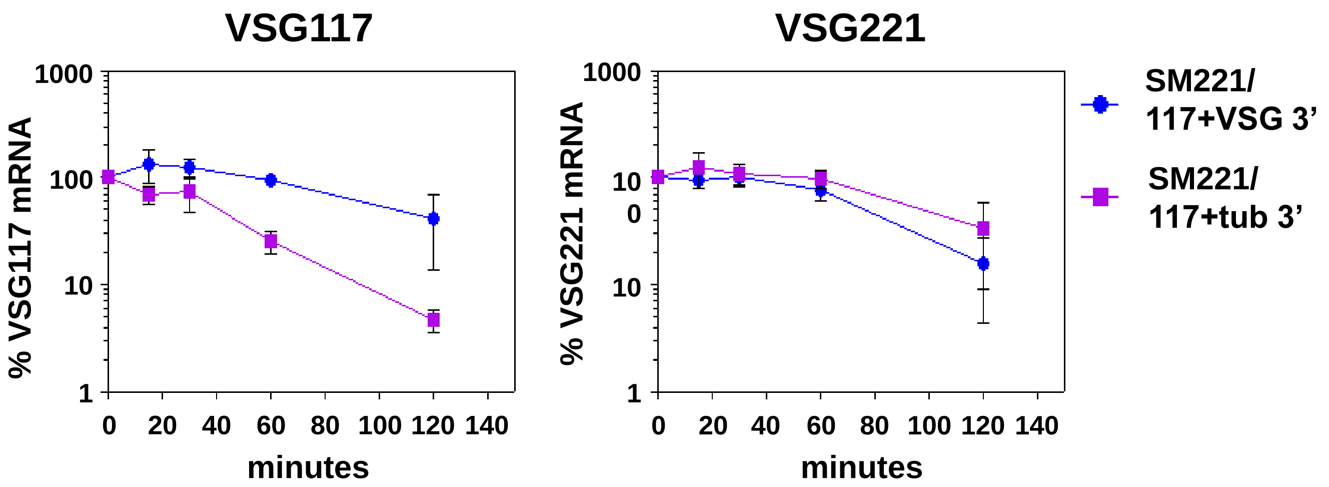

B

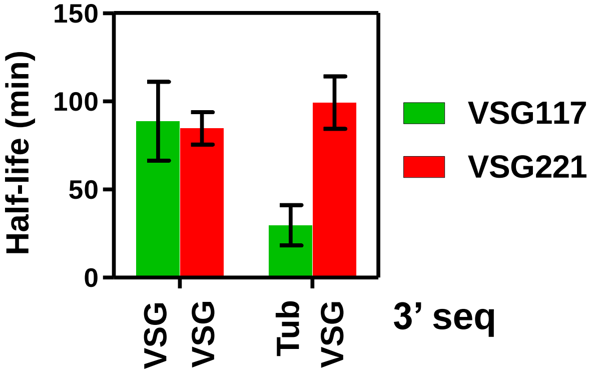

C

Half-life (min)

Cell line	VSG117	VSG221
SM221/ 117+VSG 3’	88.7 ± 22.4	84.7 ± 9.1
SM221/ 117+Tub 3’	29.7 ± 11.4	99.3 ± 14.9

Sup Fig S2

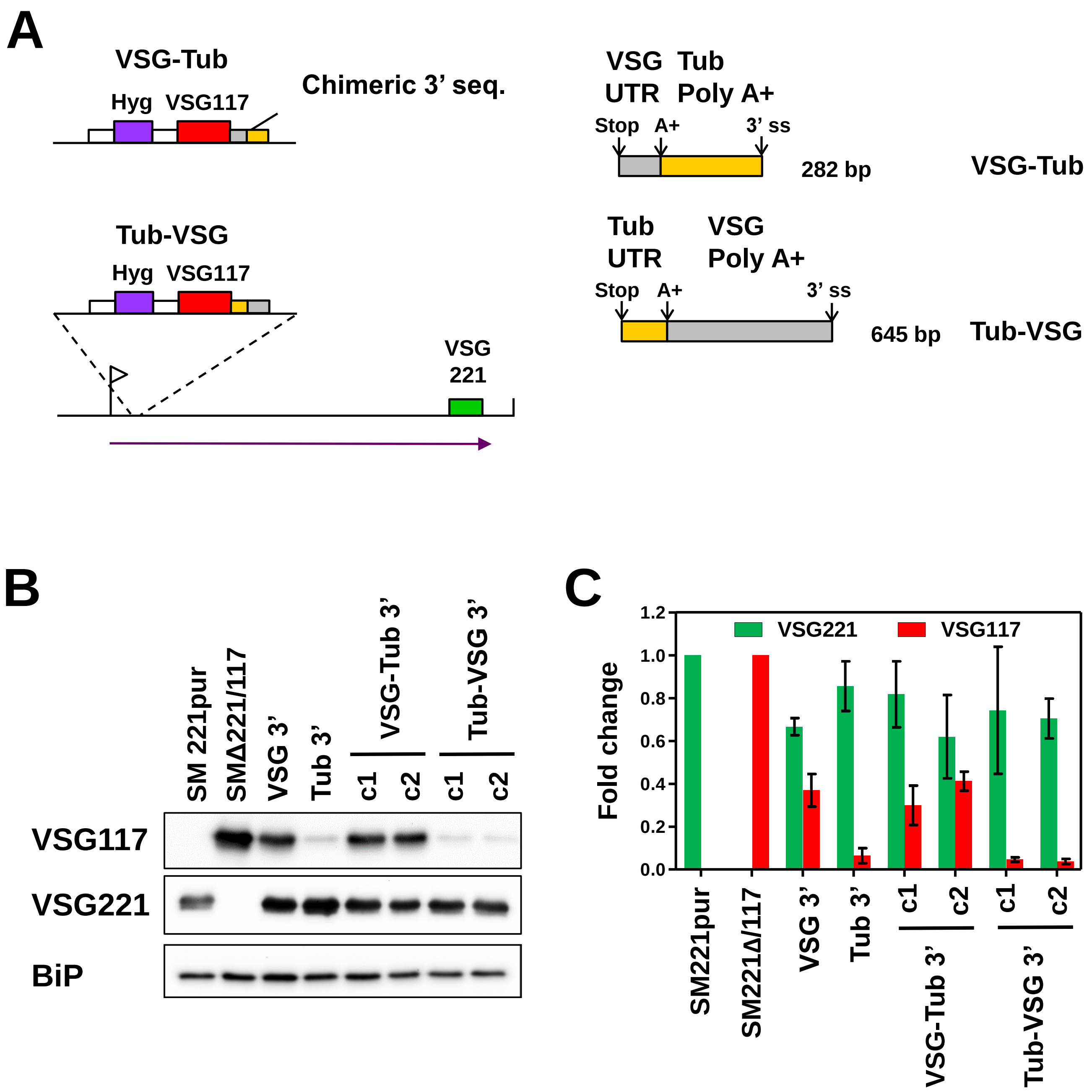
A
VSG-Tub
Hyg VSG117
Chimeric 3' seq.
VSG UTR
Tub Poly A+
Stop A+ 3' ss
282 bp
VSG-Tub
Tub-VSG
Hyg VSG117
Tub UTR
VSG Poly A+
Stop A+ 3' ss
645 bp
Tub-VSG
VSG 221
B
SM 221pur
SMΔ221/117
VSG 3'
Tub 3'
VSG-Tub 3'
Tub-VSG 3'
c1 c2 c1 c2
VSG117
VSG221
BiP
C
VSG221
VSG117
Fold change
1.2
1.0
0.8
0.6
0.4
0.2
0.0
SM221pur
SM221Δ/117
VSG 3'
Tub 3'
c1 c2 c1 c2
VSG-Tub 3'
Tub-VSG 3'

Sup Fig S3

VSG

20 40 60

TAGcccggggatccctgcagccctttccccctcaaatttccccctccttttaaattttccttgctacttgaa

80 100

aactttttgatatattttaacaccaaaacca

VSG-tub

20 40 60

TAGcccggggatccctgcagccctttccccctcaaatttccccctccttttaaattttccttgctacttga

80 100 120 140

aaactttttgatatattttaacaccaattcgcttgggacctatgtttttcttgtttttttgctcaccctttgtgt

160 180 200

aggaggcaccctgtcacgtctgtggttgcgtgtatgccttccttccccttattcgct

tub

20 40 60

TAGcccggggatccctagaaagtgtgacaacgtcgcaccatgtgtaggttttcatttatgttctttctttctttc

80 100 120 140

ttttgtgaatttgttttctgtctcaaatgtttttaattcgcttgggacctatgtttttcttgtttttttgctcacc

160

ctttgtgtagga

tub-VSG

20 40 60

TAGcccggggatccctagaaagtgtgacaacgtcgcaccatgtgtaggttttcatttatgttctttctttctttct

80 100 120

tttgtgaatttgttttctgtctcaaatgttttttaaaaccagccgagattttgt

Sup Fig S4

VSG 3' UTR

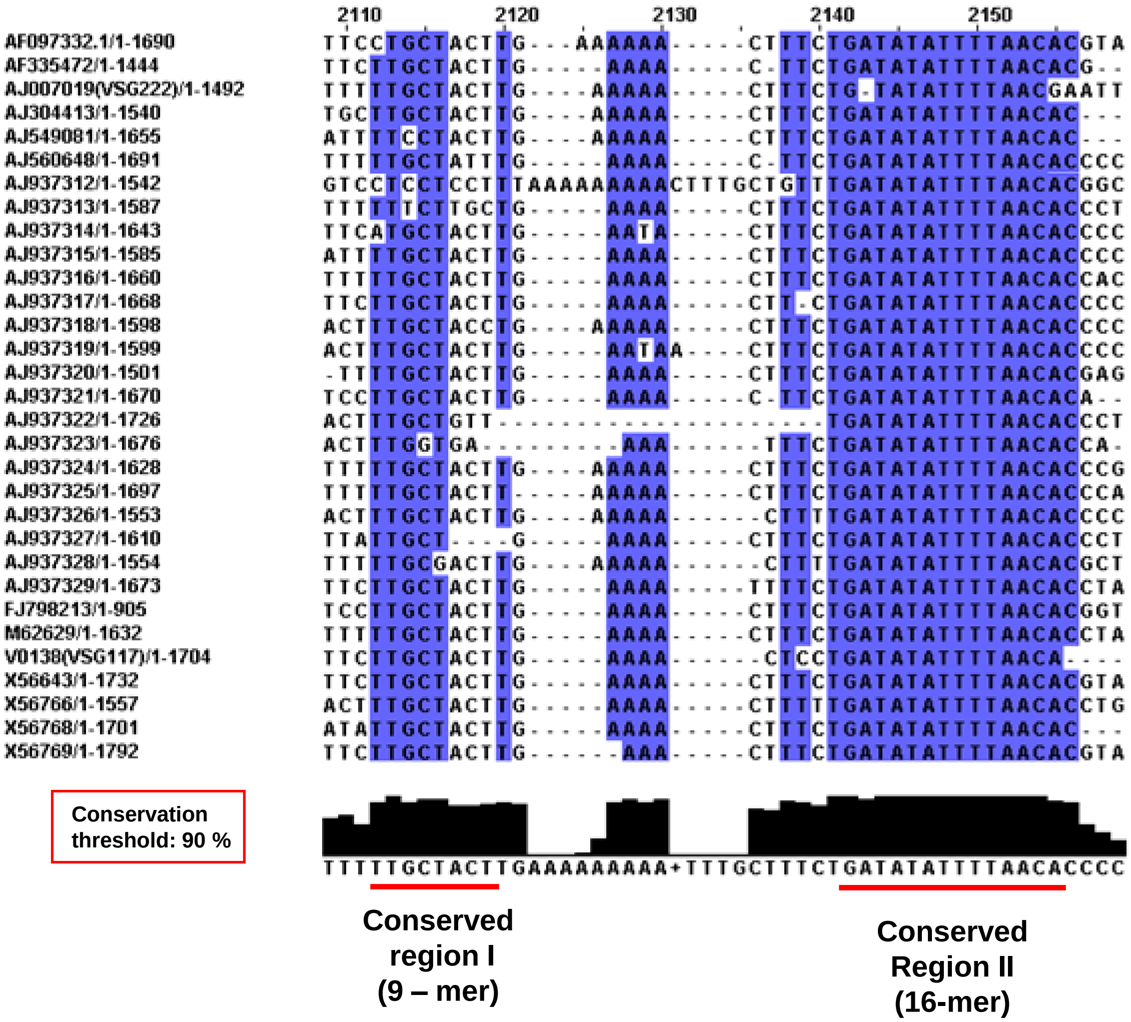

Sup Fig S5

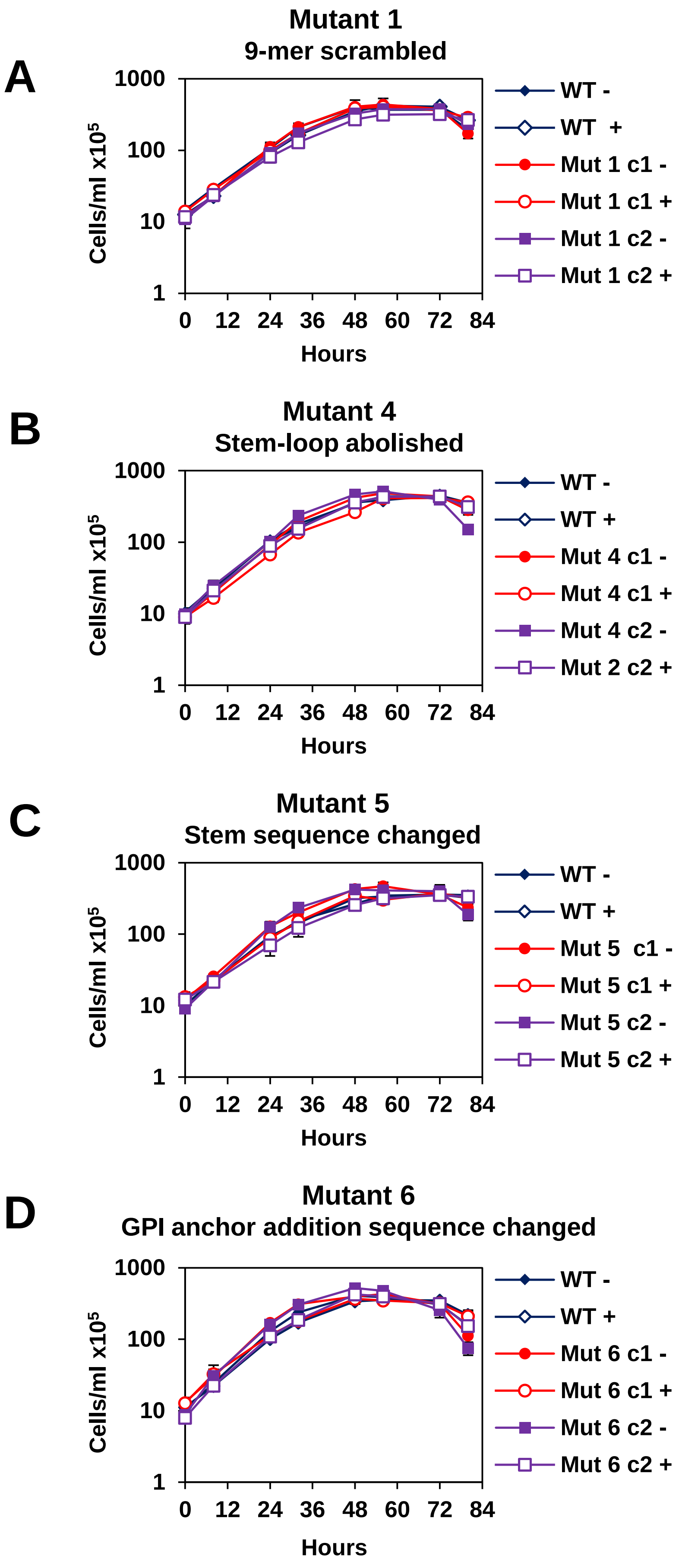
A
Mutant 1
9-mer scrambled
Cells/ml x10^5
1000
100
10
1
0 12 24 36 48 60 72 84
Hours
WT -
WT +
Mut 1 c1 -
Mut 1 c1 +
Mut 1 c2 -
Mut 1 c2 +
B
Mutant 4
Stem-loop abolished
Cells/ml x10^5
1000
100
10
1
0 12 24 36 48 60 72 84
Hours
WT -
WT +
Mut 4 c1 -
Mut 4 c1 +
Mut 4 c2 -
Mut 2 c2 +
C
Mutant 5
Stem sequence changed
Cells/ml x10^5
1000
100
10
1
0 12 24 36 48 60 72 84
Hours
WT -
WT +
Mut 5 c1 -
Mut 5 c1 +
Mut 5 c2 -
Mut 5 c2 +
D
Mutant 6
GPI anchor addition sequence changed
Cells/ml x10^5
1000
100
10
1
0 12 24 36 48 60 72 84
Hours
WT -
WT +
Mut 6 c1 -
Mut 6 c1 +
Mut 6 c2 -
Mut 6 c2 +

Sup Fig S6

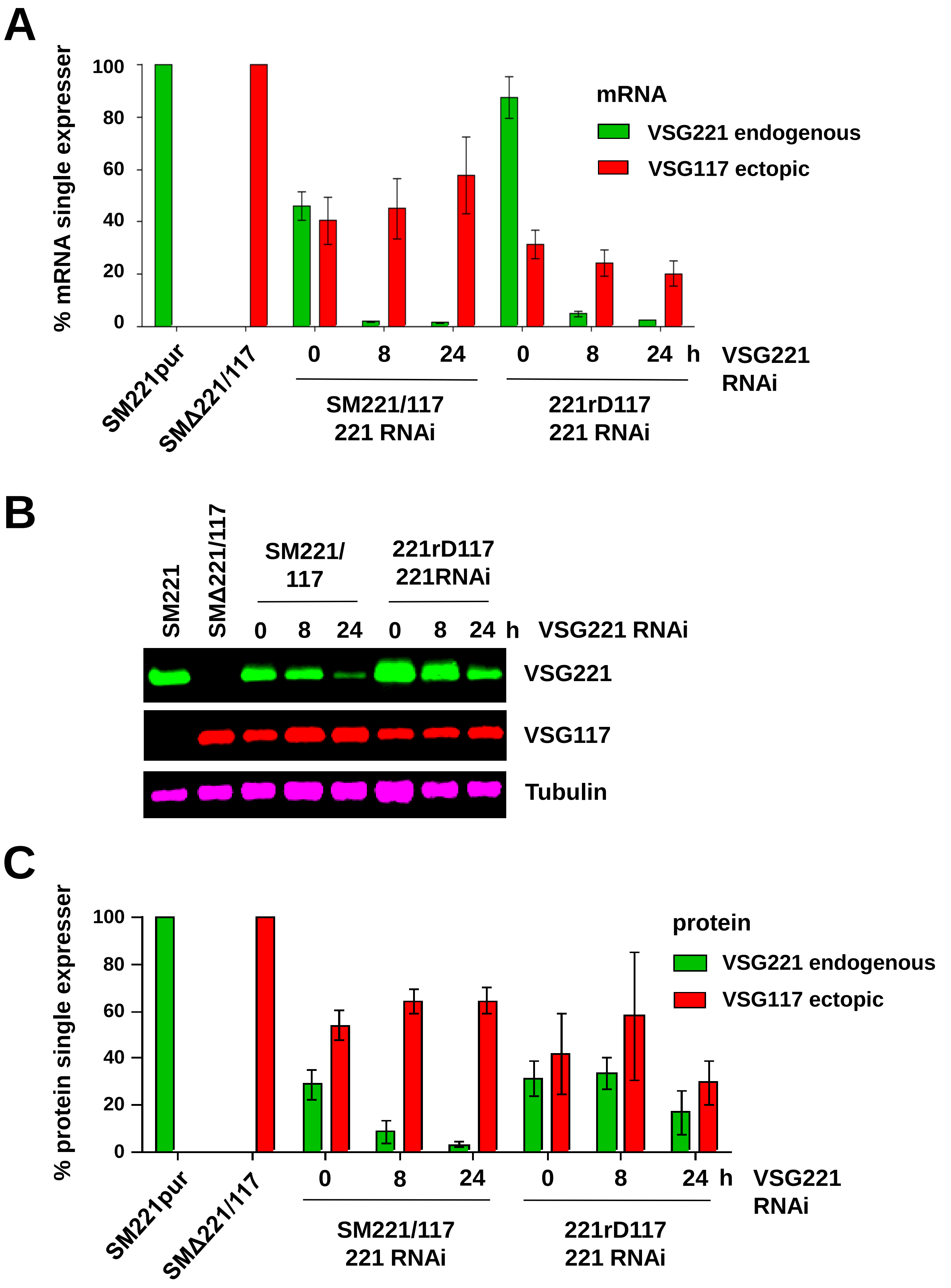
A
% mRNA single expresser
100
80
60
40
20
0
mRNA
VSG221 endogenous
VSG117 ectopic
SM221pur
SMΔ221/117
0
8
24
0
8
24 h
VSG221 RNAi
SM221/117 221 RNAi
221rD117 221 RNAi
B
SM221
SMΔ221/117
SM221/ 117
221rD117 221RNAi
0
8
24
0
8
24 h
VSG221 RNAi
VSG221
VSG117
Tubulin
C
% protein single expresser
100
80
60
40
20
0
protein
VSG221 endogenous
VSG117 ectopic
SM221pur
SMΔ221/117
0
8
24
0
8
24 h
VSG221 RNAi
SM221/117 221 RNAi
221rD117 221 RNAi

Sup Fig S7

Figure legends Supplementary Figures

Sup. Fig. S1

Inverse correlation between levels of *VSG117* and *VSG221* when ectopic *VSG117* is expressed at different levels.

Inverse correlation of mean *VSG117* and *VSG221* mRNA levels ($2^{-\Delta Ct}$) in the cell lines used in this study. A total of 32 cell lines are included in this analysis (blue dots). These cell lines include those used to analyse the effect of genomic location on expression of ectopic *VSG117* as well as cell lines where the *VSG* 3'UTR is mutated. The VSG117 and VSG221 single expressers are also included (red dots). Linear regression analysis is shown, with the value for the level of either *VSG117* or *VSG221* shown as $2^{-\Delta Ct}$.

Sup. Fig. S2

VSG117 transcript with a *VSG* 3'UTR has a three-fold higher half-life than *VSG117* with a tubulin 3'UTR.

A. The rate of decay of *VSG* transcript in cells expressing ectopic *VSG117* with either *VSG221* or α-tubulin 3' sequences was monitored over time using qPCR. *T. brucei* SM221/117 *VSG* 3'UTR or *T. brucei* SM221/ 117 tub 3'UTR cell lines were incubated with sinefungin to block trans-splicing and actinomycin D to inhibit transcription. *VSG* transcript levels were determined using qPCR expressed relative to levels in cells expressing only *VSG117* or *VSG221*.

B. The half-life in minutes (min) of transcript from the ectopic *VSG117* flanked by either *VSG* or tubulin 3' sequences compared with transcript from endogenous *VSG221* in *T. brucei* SM221/ 117 + *VSG* 3'UTR or *T. brucei* SM221/ 117 tub 3'UTR cell lines.

C. The half-life in minutes (min) of *VSG117* or *VSG221* transcript in the *T. brucei* SM221/117 *VSG* 3'UTR or *T. brucei* SM221/117 Tub 3'UTR cell lines. The half-lives were calculated with Prism software using a sigmoidal nonlinear regression curve. Results shown are the mean of at least three independent experiments with standard deviation indicated with error bars.

Sup. Fig. S3

A. Generation of *T. brucei* SM221/117 cell lines with ectopic copies of *VSG117* flanked downstream by chimeric sequences (SM221/117 VSG-Tub3' and SM 221/117 Tub-VSG3') inserted immediately downstream of the promoter of the active *VSG221* ES (as in Fig. 1). The chimeric 3' sequences indicated in panel A. are composed of the *VSG221* 3'UTR (grey box) linked to sequences downstream of the α-tubulin polyadenylation site (orange box) (VSGTub). Alternatively, the tubulin 3'UTR (orange box) is linked with sequences downstream of the *VSG221* polyadenylation site (grey box) (Tub VSG). The predicted polyadenylation site (Poly A) is shown in addition to the stop codon and predicted downstream 3' splice site (3' ss).

B. High levels of VSG expression are dependent on a *VSG* 3'UTR . Western blot analysis was performed using protein lysates from 'single-expressers' *T. brucei* SM221pur (VSG221+) or *T. brucei* SMΔ221/117 (VSG117+). Expression of ectopic *VSG117* flanked by downstream sequences from either *VSG* (*VSG* 3') or tubulin (Tub 3') was compared with expression of *VSG117* flanked downstream with either the VSG-tub or tub-VSG chimeric downstream regions. Two clones (c1 or c2) were analysed for each of these chimeric cell lines. The blot was probed for VSG221, VSG117 or BiP as a loading control.

C. Quantitation of *VSG117* or *VSG221* transcript levels for the cell lines shown in panel B. Results were normalised against actin and expressed as a percentage of the relevant single expresser cell line SM221pur (VSG221+) and SMΔ221/117 (VSG117+). The results are the mean of three independent experiments with the standard deviation indicated with error bars.

Sup. Fig. S4

3' RACE was used to identify the polyadenylation sites used when the *VSG117* gene was flanked downstream with 3' sequences from *VSG* or tubulin, or 3' sequences which were chimeric: *VSG*-tubulin (VSG-Tub) or tubulin-VSG (Tub-VSG). Two clones (c1 and c2) were analysed for each of these cell lines. RNA was isolated, and cDNA generated using a gene specific primer and a primer containing an oligodT sequence. 3' UTRs were amplified using nested PCR, cloned and sequenced. The nucleotide position in the UTR used (Pos. 3'UTR), as well as frequency (Freq.) is indicated. The frequency of usage of the different polyadenylation sites is indicated in Sup. Table 1.

Sup. Fig. S5

Alignment of 3'UTR sequences from *T. brucei VSG* cDNAs highlighting the 9-mer and 16-mer conserved regions.

Sequence alignments are shown as a Jalview presentation of the alignment as determined using MUSCLE (Multiple Sequence Comparison by Log-Expectation, EMBL-EBI). The NCBI protein sequence accession numbers are indicated on the left. The analysis included cDNA sequences from *T. brucei* field isolates (Hutchinson *et al.*, 2007), with a conservation threshold between sequences set to 90% homology.

Sup. Fig. S6

Mutations of the *VSG* 3'UTR that do not disrupt the conserved 16-mer region do not affect the ability of ectopic *VSG117* to functionally complement the cell.

Growth curves were performed using *T. brucei* cell lines expressing ectopic *VSG117* with either a wild type (WT) or a mutated *VSG* 3'UTR with mutations 1 and 4-6 as described

in Fig. 5 with sequences shown in Sup. Table 2. *VSG221* RNAi was induced in the presence (+) or absence (-) of tetracycline to test the ability of ectopic *VSG117* to complement the loss of VSG221 over time. Results are the mean of three independent experiments with standard deviation indicated with error bars.

Sup. Fig. S7

Quantitation of levels of expression of ectopic *VSG117* located in either the active *VSG221* ES (SM221/117 221 RNAi) or in an rDNA spacer (221rD117 221 RNAi).

A. Transcript levels of *VSG221* or *VSG117* were quantitated using qPCR after the induction of *VSG221* RNAi for the time indicated in hours (h). Transcript levels are expressed as the percentage (%) of the levels obtained from the 'single-expressers' SM221pur (VSG221+) or SMΔ221/117 (VSG117+) respectively. Results are from three biological replicates with standard deviation indicated with error bars.

B. Levels of VSG117 and VSG221 protein after the induction of *VSG221* RNAi for the time indicated in hours (h) as analysed using LiCor. Levels of VSG from the 'single-expressers' SM221pur or SMΔ221/117 are shown in comparison with tubulin serving as a loading control.

C. Quantitation of LiCor analysis of VSG levels expressed as percentage (%) of protein expressed from the relevant 'single-expresser' SM221pur or SMΔ221/117. Data was obtained from three biological replicates, with the standard deviation indicated with error bars.

Supplementary Table 1

Analysis of RNA from ectopic VSG117 flanked with chimaeric VSG or tubulin 3’ regions using 3’RACE.

The position of the polyadenylation site is indicated for the constructs as numberd from the stop codon and shown in Sup. Fig. S3.

VSG 3’UTR	Position 3’UTR	Freq.
**VSG**	97	9/9
**VSG-tub c1**	97	6/10
	111	1/10
	113	2/10
	197	1/10
**VSG-tub c1**	97	10/11
	129	1/11
**tub**	107	2/11
	123	8/11
	154	1/11
**tub-VSG c1**	107	10/12
	118	1/12
	120	1/12
**tub-VSG c2**	107	8/11
	118	2/11
	120	1/11

Supplementary Table 2

Sequences of the VSG 3'UTR mutants with mutated nucleotides shown in red.

Sequences shown include the last four codons of the VSG117 open reading frame and 63 nucleotides of the VSG 3'UTR.

Cell line	Mutation	Sequence of VSG 3'UTR mutants
WT		TTGCTTTTTTAATTTTCCCCCTCTTTTTCTTAAAAATTCTTGCT ACTTGAAAACTCCTGATATATTTTAACACAG
Mut1	9-mer scrambled	TTGCTTTTTTAATTTTCCCCCTCTTTTTCTTAAAAATTC**GCTTA TTCT**GAAAACTCCTGATATATTTTAACACAG
Mut2	16-mer scrambled	TTGCTTTTTTAATTTTCCCCCTCTTTTTCTTAAAAATTCTTGCT ACTTGAAAACTCC**GTTATACAAAACTTTT**AG
Mut3	9-mer and 16-mer scrambled	TTGCTTTTTTAATTTTCCCCCTCTTTTTCTTAAAAATTC**GCTTA TTCT**GAAAACTCC**GTTATACAAAACTTTT**AG
Mut4	Stem-loop abolished	TTGCTTTTTTAATTTTCCCCCTCTTT**GAT**TTAAAAATTCTTGC TACTT**CAT**AACTCCTGATATATTTTAACACAG
Mut5	Sequence of stem changed, stem-loop structure maintained	TTGCTTTTTTAATTTTCCCCCTCT**AACAA**TTAAAAATTCTTGC TACTT**TTGTT**CTCCTGATATATTTTAACACAG
Mut6	Conserved sequence at 3' end of ORF mutated	**CTTCTC**TTTTAATTTTCCCCCTCTTTTTCTTAAAAATTCTTGCT ACTTGAAAACTCCTGATATATTTTAACACAG

Supplementary Table 3

Polyadenylation is not significantly affected by mutation of the VSG 3'UTR 16-mer sequence.

The polyadenylation site was determined in VSG117 transcripts where *VSG117* was flanked downstream with *VSG* downstream sequences with either a wild type 16-mer sequence, or one where the 16-mer sequence was scrambled (Mutant 2). 3' RACE was used to identify VSG117 polyadenylation (polyA) sites. RNA was extracted, and cDNA was generated with an oligo dT containing universal primer. 3' UTR regions were amplified by nested PCR, cloned and sequenced.

**Type of 3' UTR**	**Position in 3' UTR**	**Frequency**
**VSG 16-mer WT** **n=11**	61	7
	56	2
	59	1
	72	1
**VSG 16-mer scrambled Mut 2** **n=11**	61	7
	60	2
	74	1

Supplementary Table 4

Cell lines and constructs used in this study.

**Cell line**	**Construct integrated**	**Parental cell line**	**Published in**
HNI (V02)			Rudenko *et al.* 1998
SM221			Wirtz *et al.* 1999 'single marker'
SM221pur	pHNES221 Pur1.6	SM221	Stanne *et al.* 2011
SM221/117	p221ES117 pur	SM221pur	Smith *et al.* 2009
SMΔ221/117	pBS VSG221KOblast	SM221/117	
SM221/117 VSG3'UTR	p221_117_Hyg_VSG3UTR	SM221pur	
SM221/117 Tub3'UTR	p221_117_Hyg_abTub3UTR	SM221pur	
SM221/rDNA eGFP	prDNA Targ rPro eGFP BSD	SM221pur	
SM221/rDNA117VSG3'UTR	prDNAUP_rPro_Hyg_117_VSGUTR_BSDDS	SM221/rDNA eGFP	
SM221/rDNA 117Tub3'UTR	prDNAUP_rPro_Hyg_117_TubUTR_BSDDS	SM221/rDNA eGFP	
SM221/tub 117VSG3'UTR	pTub_Hyg_117_VSG3UTR	SM221pur	
SM 221/tub 117Tub 3'UTR	pTub_Hyg_117_abTub3UTR	SM221pur	
SM 221/pro 117VSG 3'UTR	pPro_rPro_Hyg_117_VSGUTR NEW DS seq	SM221pur	
SM 221/pro 117 Tub 3'UTR	pPro_rPro_Hyg_117_TubUTR	SM221pur	

SM 221/117 VSG-Tub 3'	p221 Hyg 117 VSG-abTub chimeric 3UTR	SM221pur	
SM 221/117 Tub-VSG 3'	p221 Hyg 117 abTub-VSG chimeric 3UTR	SM221pur	
221VB			Sheader *et al.* 2005 221VB1.1 & 221VB2.1
SM 221/117 WT 3'UTR	p221_117_Pur_WT VSG 3'UTR	221VB	
SM 221/117 Mut1 3'UTR	p221_117_Pur_Mut1 VSG 3'UTR	221VB	
SM 221/117 Mut2 3'UTR	p221_117_Pur_Mut2VSG 3'UTR	221VB	
SM 221/117 Mut3 3'UTR	p221_117_Pur_Mut3VSG 3'UTR	221VB	
SM 221/117 Mut4 3'UTR	p221_117_Pur_Mut4 VSG 3'UTR	221VB	
SM 221/117 Mut5 3'UTR	p221_117_Pur_Mut5 VSG 3'UTR	221VB	
SM 221/117 Mut6 3'UTR	p221_117_Pur_Mut6 VSG 3'UTR	221VB	
221VP117			Smith *et. al*., 2009
SL221	pLEW100.v5x.PEX11.VSG221RNAi	SM221pur	stem loop construct from Silverman *et. al*., 2011
221rD117 221 RNAi	pLEW100.v5x.PEX11.VSG221RNAi	SM221/rDNA 117 VSG3'UTR	stem loop construct from Silverman *et. al*., 2011

Supplementary Table 5

Primers used for cloning

Amplified feature	Template	Primer Name	Primer Sequence (5’-3’)
Downstream procyclin targeting fragment	pEP1eGFP RPtubBLAST	ProDS NotIs	CCCGCGGCCGCCCTTGGCAGCCCAATAAAC
		NewProDS SacIas	CCCGAGCTCGTTGTTTCCCCCTCTTCCTC
Upstream procyclin targeting fragment	pEP1eGFP RPtubBLAST	ProUP KpnIs	CCCGGTACCGGTTGTGTGGTAGTCGTGCG
		ProUP XhoIas	CCCCTCGAGCCGAAAGGCGAGCCCGATCC
rDNA promoter sequence	rDNA eGFP RPtubBLAST	rDNAPro_XhoIs	CCCCTCGAGCTTTCCACCCAGCGCGGGTGC
		rDNAPro_HindIIIas	CCCAAGCTTCTGTGAAGGTAATTAAAAACC
Alpha beta Tub Intergenic regions and eGFP	p221_eGFP_Pur	abTubIR EcoRI s	CCCGAATTCCCTAGAAAGTGTGACAACG
		eGFP BamHI as	CCCGGATCCTTACTTGTACAGCTCGTCC
PFR1 Intergenic Region, BSD and Actin Intergenic Region	PFRBlastAct	PFR1IR BamHI s	CCCGGATCCTGTGGCCGCAATTATT
		ActIR NotI as	CCCGCGGCCGCTATTTTATGGCAGCAACG
Blasticidin gene sequence	pGad8 Tubulin GFP (Blast)	BSD Targ NotIs	CCCGCGGCCGCGCCAAGCCTTTGTCTCAAG
		BSD Targ SacIas	CCCGAGCTCTTAGCCCTCCCACACATAACC
Upstream	rDNA eGFP RPtubBLAST	rDNATF_KpnISacIs	GAGGGTACCGAGCTCGCGAGGCGAATCGCTCAGTAG

rDNA targeting fragment		rDNTrg_NewX_s	CTG CTCGAGCTGATGGCATGCCAATTTCACTAC
Downstream rDNA targeting fragment	rDNA eGFP RPtubBLAST	rDNATarg_NotIs	GATAGCGGCCGCTATCGATAAAAATGATACG
		rDNATarg_SacIas	GATGAGCTCCATATAGTTGGTATGTATTCTAATTCC
Beta-alpha tubulin IR	*T. brucei* genomic DNA	baTubIR_KpnIAscIs	CCCGGTACCGGCGCGCCCGCGGACGGGGCATTTCCCG
		baTubIR_BglIIas	CCCAGATCTGTCAGAAATCAGCACCGCG
Alpha-beta Tub3UTR and Tubulin downstream Targeting Fragment	*T. brucei* genomic DNA	abTub3UTR_BamHIs	GATGGATCCCTAGAAAGTGTGACAACGTC
		BetaTub_SacI626as	CCCGAGCTCTCGTACAGTGCCTCGTTGTC
Tubulin downstream Targeting Fragment	*T. brucei* genomic DNA	abTubIR2_NotIs	CCCGCGGCCGCCCCTTATTCGCTTCTTCCTG
		BetaTub_SacI626as	CCCGAGCTCTCGTACAGTGCCTCGTTGTC
221 KO upstream targeting fragment	*T. brucei* genomic DNA	221KO_up56363s	GCGTCTAGATGCAGCATATGTAACTACCTCTCG
		221KO2_up57045as	GCTGGATCCTGTCGCGTAGGAATAACTACAATC
221 KO downstream targeting fragment	*T. brucei* genomic DNA	221KO_dn58200s	CCGAAGCTTCTATTACCGAATGGAAACAGCAG
		221KO2_dn58775as	CGCCTCGAGTCCTAAGCCTAAGTCTAATCCTAATCC
VSG221 3’UTR + downstream sequence	*T. brucei* genomic DNA	VSGUTR_BamHIs	GATGGATCCCTGCAGCCCTTTCCCCCCTCAA
		VSGUTR_NotIas:	GATGCGGCCGCCCCCGAAAAATTAAGATTCA
Chimeric VSG-Tub 3’UTR	p221_117+VSG 3’UTR/ p221_117+Tub 3’UTR	VSG-Tub Antisense chimeric primer	GAAAAACATAGGTCCCAAGCGAATTGGTGTTAAAATATATCAAAAAG

		VSGUTR_BamHIs	GATGGATCCCTGCAGCCCTTTCCCCCCTCAA
		abTub3UTR_NotIas	GATGCGGCCGCAGTAGGGCTGGTCGAGAAAAC
Chimeric Tub-VSG 3’UTR	p221_117+VSG 3’UTR/ p221_117+Tub 3’UTR	Tub-VSG Antisense chimeric primer	GAACACAAAATCTCGGCTGGTTTTAAAAACATTTGAGACAGAAAAC
		abTub3UTR _BamHIs	GATGGATCCCTAGAAAGTGTGACAACGTC
		VSGUTR_NotIas	GATGCGGCCGCCCCCGAAAAATTAAGATTCA
Stem-loop *VSG221* RNAi construct	*T. brucei* genomic DNA	SLS1	TTTTTTAAGCTTTTGGTAACGCCTGTTTTGCC
		SLAS1	TTTTTTCTCGAGTTTGGCAACCTCTTTGCCAG
		SLS2	TTTTTTTGGATCCTTGGTAACGCCTGTTTTGCC
		SLAS2	TTTTTTTCTAGATTTGGCAACCTCTTTGCCAG

Supplementary Table 6

Primers used for qPCR and RACE

**qPCR primers**		
**Target**	**Forward Primer**	**Reverse Primer**
VSG117	GTCACACAATGGAGGCGATC	TCCGCAGTGGTTTGTCCAAG
VSG221	GCGACAACCAGCCAACCAAG	TCAGCGGGCTTGTGCTTCTG
Actin	GTTCCATCCTCTCATCACTA	TCGTATTCACTCTTCGTTATC
rDNA 28S beta	GTAAGTTCGCAAGAAGCAT	ACCAGAAGGAGGTTAGTAGATA
VSG221 ES *pseudogene*	GCAGGCAAGCATTACCAGAG	CTGTTCCGAATAGCGCGTC
VSG221 ES co-transposed region	TGGAGCGTACACACAAGTGA	ATGCATTGGCACACTTTCCG
**3'RACE primers**		
Q_T	CCAGTGAGCAGAGTGACGAGGACTCGAGCTCAAGCTTTTTTTTTTTTTTTTT	
Q_0	CCAGTGAGCAGAGTGACG	
Q_1	GAGGACTCGAGCTCAAGC	
VSG117 primer 1	CGGCGACCAAAGGCAGCACGAAAGAG	
VSG117 primer 2	GTGCTGCTGCGTTTGCGGCCTTGCTT	
